# Traditional knowledge of childhood illnesses among the Sidama nation, clinical presentation and hematological findings

**DOI:** 10.1371/journal.pone.0340246

**Published:** 2026-11-10

**Authors:** Serawit Deyno, Mairegu Daniel, Tsadiku Getachew, Menfese Tadesse, Hizkel Engiso, Sintayehu Fekadu, Nigatu Tuasha

**Affiliations:** 1 School of Pharmacy, Hawassa University, Hawassa, Ethiopia; 2 Kampala International University, Ishaka, Bushenyi District, Uganda; 3 Department of Chemical Engineering, Hawassa University, Hawassa, Ethiopia; 4 Adaare General Hospital, Hawassa, Ethiopia; 5 Soil Resources and Watershed Management, Wondo Genet College of Forestry and Natural Resources, Hawassa University, Hawassa, Ethiopia; 6 School of Medical Laboratory technology, Hawassa University, Hawassa, Ethiopia; 7 Biomedical Sciences, Department of Biology, Hawassa College of Teacher Education, Hawassa, Ethiopia; Kwame Nkrumah University of Science and Technology Faculty of Pharmacy and Pharmaceutical Sciences, GHANA

## Abstract

**Background:**

Sidama possesses extensive indigenous knowledge regarding infant illnesses described as “*feyancho*”. Feyancho has a widespread recognition among Sidama people, though poorly documented in scientific literature. This study aimed to document the indigenous understanding of this infantile disease and examine associated clinical and biochemical profiles in the Sidama National Regional State, Ethiopia.

**Methods:**

A household-based cross-sectional study was conducted in nine districts selected using systematic random sampling method. Data was collected through household surveys (n = 436), focus group discussions (FGDs), and clinical assessments. Clinical and biochemical evaluations were performed on children affected by” feyancho” disease.

**Result:**

The overall prevalence of “*feyancho*” was 59.52%. Most respondents (66.51%) believed that maternal health influences the occurrence of “feyancho” disease. In the study group, liver function tests showed elevated levels of SGOT (AST), ALP, and direct bilirubin in more than half of the children. Additionally, 25% presented low glucose and elevated total bilirubin. Hematological analyses revealed significant increases in LYM (+128%), EOS (+76.92%), LYM% (+79.6%), RDW-CV (+36.23%), PDW (+35.74%), and PCT (+1015.79%). Conversely, decreases were observed in NEU (−29.05%), NEU% (−36.78%), RBC (−13.26%), HGB (−28.07%), HCT (−29.36%), MCV (−20.73%), and PLT (−25.67%).

**Conclusion:**

The Sidama people’s indigenous knowledge of infantile diseases remains an integral part of local health practices. Clinical and biochemical findings suggest conditions such as microcytic anemia, inflammation, or chronic infections. Safeguarding this traditional knowledge and validating it scientifically is crucial, requiring more comprehensive research.

## Introduction

Indigenous knowledge systems (IKS) play a crucial role in the understanding childhood diseases and have been used for generations to diagnose and treat illnesses [1,2]. The Sidama nation commonly defines infantile diseases as “*Feyancho*”. The “*feyancho*” can be either a milder form, locally called “*Sidancho*”, or a severe form, also locally known as “*Arusicho*”. This study is the first of its kind in attempting to document this indigenous knowledge on community understanding of diseases, providing a foundation for future research in the field. The finding of the study will be used as a starting point for the integration of IKS on identifying diseases and aligning to modern medicine understanding and treatment.

Indigenous communities follow a holistic approach to diagnosing and managing infantile illnesses, integrating cultural beliefs, environmental observations, and generational knowledge. Unlike biomedical methods that focus on specific pathogens, traditional diagnosis relies on symptom-based assessments, spiritual interpretations, and behavioural changes. Infantile illnesses are often identified through signs such as appetite loss, restlessness, body temperature fluctuations, or digestive disturbances [3].

Studying traditional understanding of infantile diseases is crucial to have a clear picture of the merits and demerits. Understanding these practices enables healthcare providers to foster trust, bridge cultural gaps, and integrate effective remedies into formal healthcare, ultimately enhancing infant health outcomes while preserving cultural heritage [4].

There has been no scientific investigation into how these diseases are understood by the local community. To fill this gap, the present study aimed to document the IKS related to “*feyancho*” among selected rural communities in the Sidama National Regional State (SNRS) and explored how people in the study area understand this infant disease. This case study also examined the study participants clinically and biochemically.

## Materials and methods

### Study design

A community-based cross-sectional mixed-methods study was conducted from January to December 2023 in the Sidama National Regional State (SNRS), Ethiopia. The study integrated quantitative household surveys, qualitative Focus Group Discussions (FGDs), and clinical, biochemical, and hematological assessments to comprehensively document indigenous knowledge related to the infantile disease locally known as “feyancho” and to examine associated clinical and laboratory findings. The study consisted of two distinct components:

- A community-based cross-sectional survey involving 436 participants to document Indigenous knowledge, perceptions, and practices related to Feyancho.
- A clinical case series involving 13 infants diagnosed with Feyancho during the study period, in which clinical symptoms are examined and among which12 have consented for blood samples collection for hematological and biochemical analyses.

The protocol of this study is supplied Supporting Information S1 File.

### The study area

The study was conducted in nine (9) Woredas (Districts) of the SNRS stratified based on the agro-climatic zones of the region. Two kebeles (the smallest administrative unit) were randomly selected from each woreda. Three different agro-climatic zones exist in Sidama [5]. These are “*Kolla*”, “*Woina Dega*” and “*Dega*” agro-climatic zones. ‘*Kolla’* is characterized by a dry, hot tropical climate area and lies between 500 and 1500 meters above sea level. It receives an annual rainfall of 400–800 mm, with a mean annual temperature of 20–25 °C and constitutes 30% of the total area. The ‘*Woina Dega*’ is characterized by a moist to humid, warm subtropical climate and is found between 1500–2500 meters above sea level, with an annual rainfall of 1000–1800 mm. This agro-climatic zone has a mean annual temperature of 15–20 °C and constitutes 54% of the total area of the region. The ‘Dega’ agro-climatic zone is known with its wet, cool temperate climate. “*Dega*” has an elevation of 2500–3500 meters above sea level and receives an annual rainfall of 1200–1800 mm, and the mean annual temperature is 10–15 °C and constitutes only 16% of the total area of the region [6].

The nine woredas included in the present study were Borricha, Daraara and Hawassa Zuria woredas representing *Qolla’* (dry, hot tropical climate); Haweela, Daalle, Daarra woredas from *Woina Dega’* (moist to humid, warm subtropical climate) and Hula, Bursa and Arbagoona from *Dega’* (wet, cool temperate climate). From each of the selected districts, two kebeles (a total of 18 kebeles) were randomly selected for the study. The sampling units are households from the selected kebeles. The study area is presented in Fig 1.

**Fig 1:**
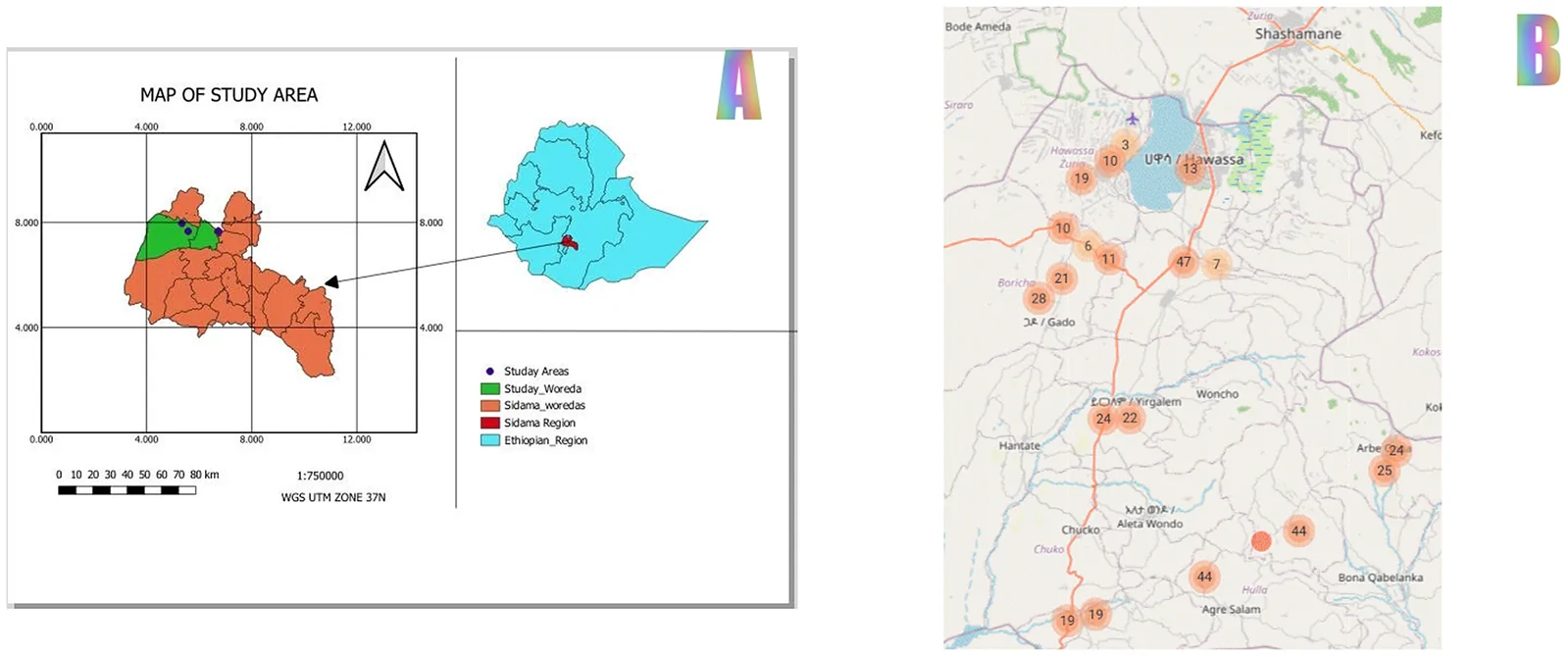
Map showing the woredas (districts) included in the study from Sidama National Regional State, Ethiopia. “A” drawn using QGIS; administrative boundaries are from geoBoundaries (https://www.geoboundaries.org/), used under Creative Commons Attribution 4.0 International License. “B” generated from Kobo Collect using Leaflet with OpenStreetMap base map tiles (OpenStreetMap contributors), used under the Open Data Commons Open Database License (ODbL).

### Study population and sample collection

There is a detailed survey report based on the data collected regarding “*feyancho*” disease in SNRS, Ethiopia. The socio-demographics, understanding of diseases and symptoms of” feyancho”, perception on disease transmission and prevention were recorded. In addition, clinical samples from children affected by the “*feyancho*” were collected for biochemical analysis. Household sample units were studied to determine the prevalence of infant diseases. The prevalence of “*feyancho*” was based on the number of total births in the sampled households.

The sample size for this study was determined based on the presence of attribute in the general population which was estimated to be at 50%. Thus, the sample size was calculated based on a single population proportion formula by taking the margin of error of 0.05 at 95% confidence level (CI), the total household unit sampled as n= (Zα/2)^2^p (1-p)/d^2^. In addition, 10% of non-response rate was considered. Accordingly, a total of 436 study participants were included in the study.

The prevalence of “feyancho”, as defined by the community, in the SNRS was determined. During this investigation, people’s perceptions and definitions of “feyancho” disease were documented. Clinical characterization and hematological parameters were evaluated from clinical samples collected from infants claimed to be affected by “feyancho”.

#### Household survey participants.

The study population comprised households residing in selected kebeles of the SNRS. Household heads or primary caregivers (preferably mothers or fathers) were eligible if they had at least one child and were willing to participate. Households were excluded if no responsible adult was available at the time of data collection or if consent was not provided.

#### Clinical and laboratory participants.

Children identified by caregivers and the community as having “feyancho” (either sidancho or arusicho) were eligible for clinical examination and laboratory investigations. Written informed consent was obtained from parents or guardians prior to sample collection.

#### Sample collection and hematological analysis.

Venous blood samples were collected from children identified with “feyancho” using aseptic techniques by trained medical laboratory professionals. Approximately 2–3 mL of blood was drawn from each child and divided into appropriate collection tubes: EDTA tubes for hematological analysis and plain or serum separator tubes for biochemical analysis.

Blood samples were gently mixed to prevent clotting and transported in cold boxes to the laboratory within 2-hour time. Samples for hematological analysis were processed promptly, while serum samples were allowed to clot, centrifuged, and stored under appropriate conditions until analysis.

Hematological parameters including white blood cell count, differential counts, red blood cell indices, hemoglobin, hematocrit, platelet indices, and red cell distribution width were analyzed using a standard automated hematology analyzer following manufacturer instructions and laboratory quality control procedures. Internal quality checks were performed daily to ensure reliability and accuracy of results.

Biochemical parameters such as liver enzymes, glucose, bilirubin, urea, creatinine, and lipid profile were measured using standard clinical chemistry analyzers according to established laboratory protocols.

#### FGD design and participant selection.

Focus Group Discussions (FGDs) were conducted first to gain clearer understanding of the community, community definition of the disease’s concepts. This complemented to the household survey data and to gain in-depth insight into community perceptions, indigenous classifications, symptoms, transmission beliefs, and amelioration practices related to the infantile disease locally known as “feyancho.” Participants were selected using purposive sampling to include community members with lived experience and cultural knowledge of childhood illnesses, including parents, caregivers, elders, and individuals recognized locally for their traditional health knowledge.

#### FGD composition and setting.

A total of eight FGD sessions were conducted across selected kebeles in the study districts. Each FGD consisted of 6–10 participants, allowing for active interaction and balanced participation. Discussions were held in familiar and neutral community settings to ensure participant comfort and openness. Where necessary, groups were organized to minimize social or cultural power imbalances.

#### Data collection procedure.

FGDs were moderated by trained facilitators fluent in the local Sidama language, with assistance from note-takers. Moderators encouraged inclusive participation and guided discussions using probing questions where appropriate. Each discussion lasted approximately 60–90 minutes. All sessions were audio-recorded with participants’ consent, and detailed field notes were taken to capture non-verbal cues, group dynamics, and contextual observations.

#### Data management.

Audio recordings were transcribed verbatim and translated into English where necessary. Transcripts were checked against recordings to ensure accuracy. All personal identifiers were removed to maintain confidentiality, and data were securely stored.

#### Ethics approval and consent to participate.

A cross-sectional household level study was conducted from January 2023 to December 2023. Informed written assent (consent) was obtained from parents or guardians for the case study. The study was conducted in accordance with the guidelines required by the National Health Council of Ethiopia through Hawassa University Research Ethics Review Committee (Ref no: RERC 02,2022) in accordance with the Declaration of Helsinki.

#### Data analysis.

Data entry and validation were done using STATA and every possible data quality assurance approaches were followed throughout the study period. Descriptive statistics (percentages and frequency) are used to summarize the data.

#### FGD data analysis.

FGD data were analysed using a thematic analysis approach, following an iterative and systematic process to identify key patterns and meanings related to community understanding of “feyancho.” Data analysis was conducted manually. To ensure methodological rigor, credibility was enhanced through triangulation of FGD findings with household survey data. Dependability was supported by maintaining a clear audit trail of coding and theme development, while confirmability was strengthened through team discussions and reflexive review of interpretations.

## Results

### Socio-demographic characteristics

A total of 436 households were included from nine Woredas, with a diverse representation across the region. Each woreda was represented with about 9.86–12.16% participants from two kebeles. The respondents have varying educational backgrounds, ranging from no formal education to higher education levels. The respondents have diverse religious affiliations, but the majority identify as Christian (Protestants). The mean age of household fathers is 45.66 (SD = 16.22) years while the median age of household mothers is 39.33 (SD = 13.50) years. The mean family size is notably larger than the mean total number of children, indicating that households also include other members beyond children, such as adults, Table 1.

**Table 1:** Socio-demographic characteristics of the study participants (N = 436), 2023.

Categorical variables					
Variables	Category	N (%)	Variables	Category	N (%)
Education Level of Household mother	No formal education	188 (43.12%)	Education Level of Household father	No formal education	187 (42.89%)
	Some Primary school	184 (42.20%)		Some Primary school	133 (30.50%)
	Some Secondary school	12 (2.75%)		Some Secondary school	24 (5.50%)
	High school complete	22 (5.05%)		High school complete	45 (10.32%)
	Diploma	19 (4.36%)		Diploma	18 (4.13%)
	Bachelor’s degree	4 (0.92%)		Bachelor’s degree	17 (3.90%)
	Master’s degree	1 (0.23%)		Master’s degree	1 (0.23%)
Religion of Household father	Christian-protestant	352 (80.73%)	Religion of Household mother	Christian-protestant	346 (79.36%)
	Apostolic	40 (9.17%)		Apostolic	44 (10.09%)
	Catholics	15 (3.44%)		Catholics	14 (3.21%)
	Muslim	11 (2.52%)		Muslim	11 (2.52%)
	Adventist	9 (2.06%)		Adventist	9 (2.06%)
	Christian-orthodox	6 (1.38%)		Christian-orthodox	7 (1.61%)
	Atheist	2 (0.46%)		Atheist	1 (0.23%)
	Traditional	1 (0.23%)		Traditional	1 (0.23%)
Continuous variables					
	Mean	Median	Mode	Standard Deviation	
Family Size	6.03	6.0	7.0	2.19	
No of Children	5.21	4	2	3.27	
No of Male	2.72	2	2	2.01	
No of female	2.49	2	2	1.82	

### Prevalence of the infant diseases (“feyancho”)

“Feyancho” was widely reported, with 67.2% of households indicating mild cases and over half reporting severe cases. Most participants believed transmission occurs from mother to child (57.3%) or through breastfeeding (40.4%), while fewer mentioned contact, genetic, or airborne routes. The condition was most commonly reported in infants under one year of age (50%), Table 2.

**Table 2:** Prevalence of “*feyancho*” as defined by the community among study participants (N = 436), 2023.

Variable	Category	Frequency (%)
Households have children with a mild “feyancho” (Sidancho)	Yes	275 (67.24%)
	No	134 (32.76%)
Households have children with a severe “feyancho” (Arusicho)	Yes	124 (58.22%)
	No	89 (20.41%)
Household belief on route of transmission of “feyancho”	Mother to child (MTC)	250 (57.34%)
	Breastfeeding	176 (40.37%)
	Contact	63 (14.45%)
	Genetic	41 (9.4%)
	Airborne	2 (0.46%)
Age of infants affected by “feyancho”	Up to 1 year	218 (50%)
	Up to 5 months	73 (16.74%)
	Up to 3 months	62 (14.22%)

According to the participants, *sidancho* or *arusicho* primarily affects very young children, with the highest prevalence observed in infants up to one year old, as reported by 218 respondents (50%). Within this age range, 19 respondents (4.36%) specifically identified children up to 8–10 months, while 73 respondents (16.74%) noted cases in infants between 3–5 months. Additionally, 62 respondents (14.22%) indicated that children up to 3 months were affected (Table 2). The data highlights that early infancy is the most vulnerable period for these diseases, emphasizing the need for targeted health interventions during this stage.

### Study participants’ belief on the mechanisms of transmission of the diseases

Most participants (N = 290; 66.51%) believed that a mother affected by “*feyancho*” increases the likelihood of her children being affected. In contrast, 73 respondents (16.74%) did not share this belief. These findings suggest that most participants recognize a potential hereditary or environmental link between a mother’s condition and her children’s susceptibility to “*feyancho*”.

The participants identified several possible ways through which “*feyancho*” may be transmitted. The most believed mode of transmission was direct transmission from mother to child, with 250 respondents (57.34%) supporting this view. Similarly, a significant portion of respondents (176 cases, 40.37%) believed that transmission occurs through breastfeeding, indicating concerns about the role of maternal feeding practices in the spread of the disease. Overall, the findings indicate that most participants believe maternal transmission, particularly through direct contact and breastfeeding, is the primary way “*feyancho*” spreads among children, Table 2.

### Community’s perception about the symptoms and amelioration of “*feyancho*”

Focused group discussions (FDGs) were conducted with community participants to complement the data obtained from structured interviews. A total of eight interview sessions were carried out to explore people’s understanding of the disease condition known as*”feyancho”*. The summaries of these interviews provide insights into participants’ perceptions and statements and are complementary with the interview findings.

Sidama nation categorizes “*feyancho*” into two types: *arusicho* (a severe form) and *sidancho* (a milder form). “*Feyancho”* is characterized by a range of symptoms and conditions. Severe cases are characterized by skin peeling, sunken fontanels, emaciation, and stomach issues. Milder conditions involve symptoms such as stomach-aches, diarrhoea, restlessness, and minor skin rashes. The amelioration of the conditions is characterized by purging of green diarrhoea, disappearance of rushes, good sleeping, and fontanels normalized, resumes normal breastfeeding, physical betterment and fattening, the stiffened belly begins to soften and so on.

### Symptoms and characteristics of the diseases as defined by the community

The symptoms vary in frequency, with skin rashes being the most common and hair loss the least common among those believed to be affected by the milder form of “*feyancho*” (*Sidancho*). Significant portion of individuals report abdominal pain as one of major symptoms, Fig 2.

**Fig 2:**
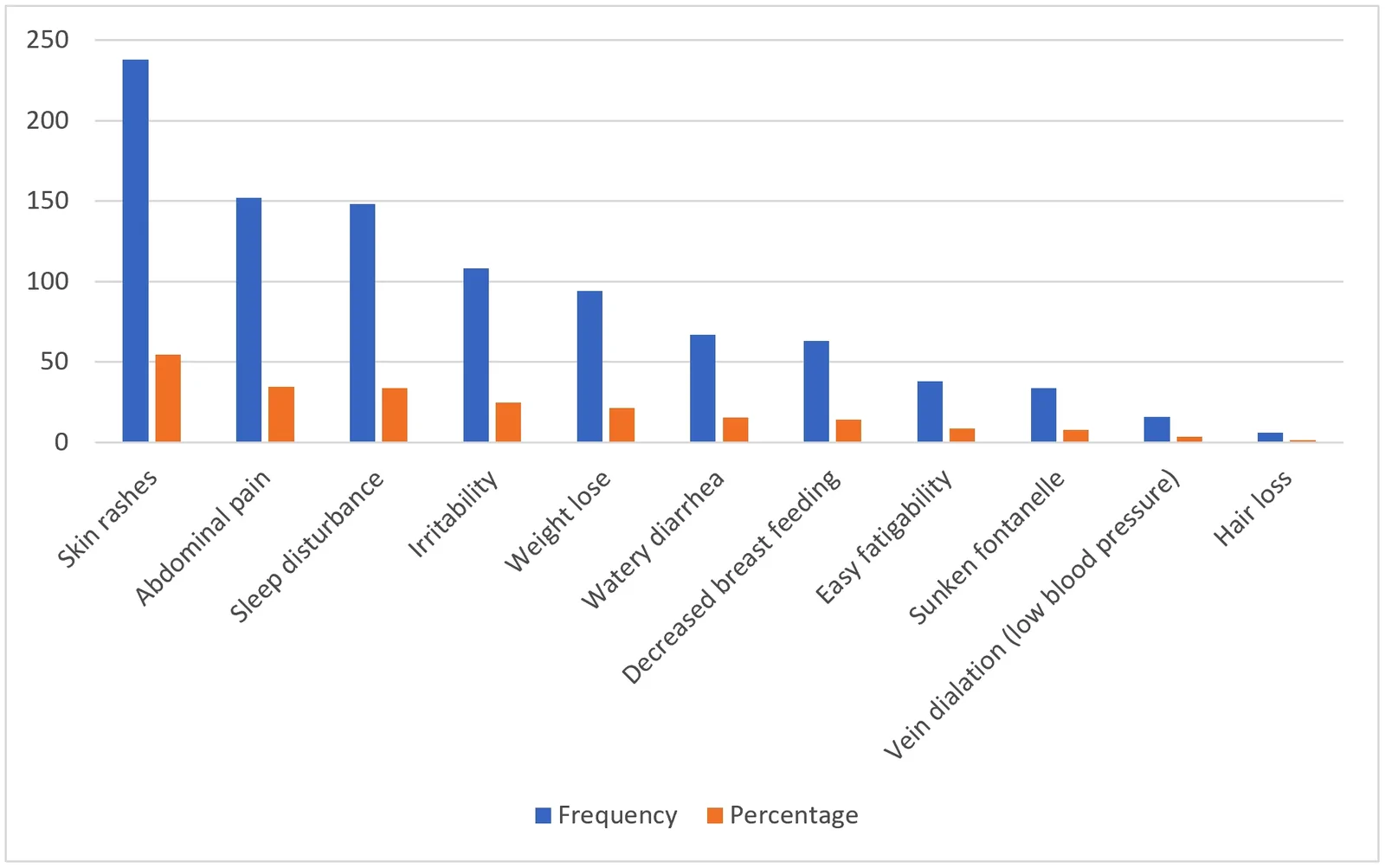
Frequency of common symptom of the mild “*feyancho*” (*Sidancho*) as described by the community.

The severe form of “*feyancho*” (*Arusicho*) is associated with several symptoms, which may include physical symptoms, psychological symptoms and cultural or spiritual implications: in some contexts, the symptoms might be interpreted as being linked to cultural or spiritual beliefs, potentially influencing the perception and treatment of the symptoms. Most reported symptom during the severe form of “*feyancho*” (*arusicho*) is that of sunken fontanelle, Fig 3.

**Fig 3:**
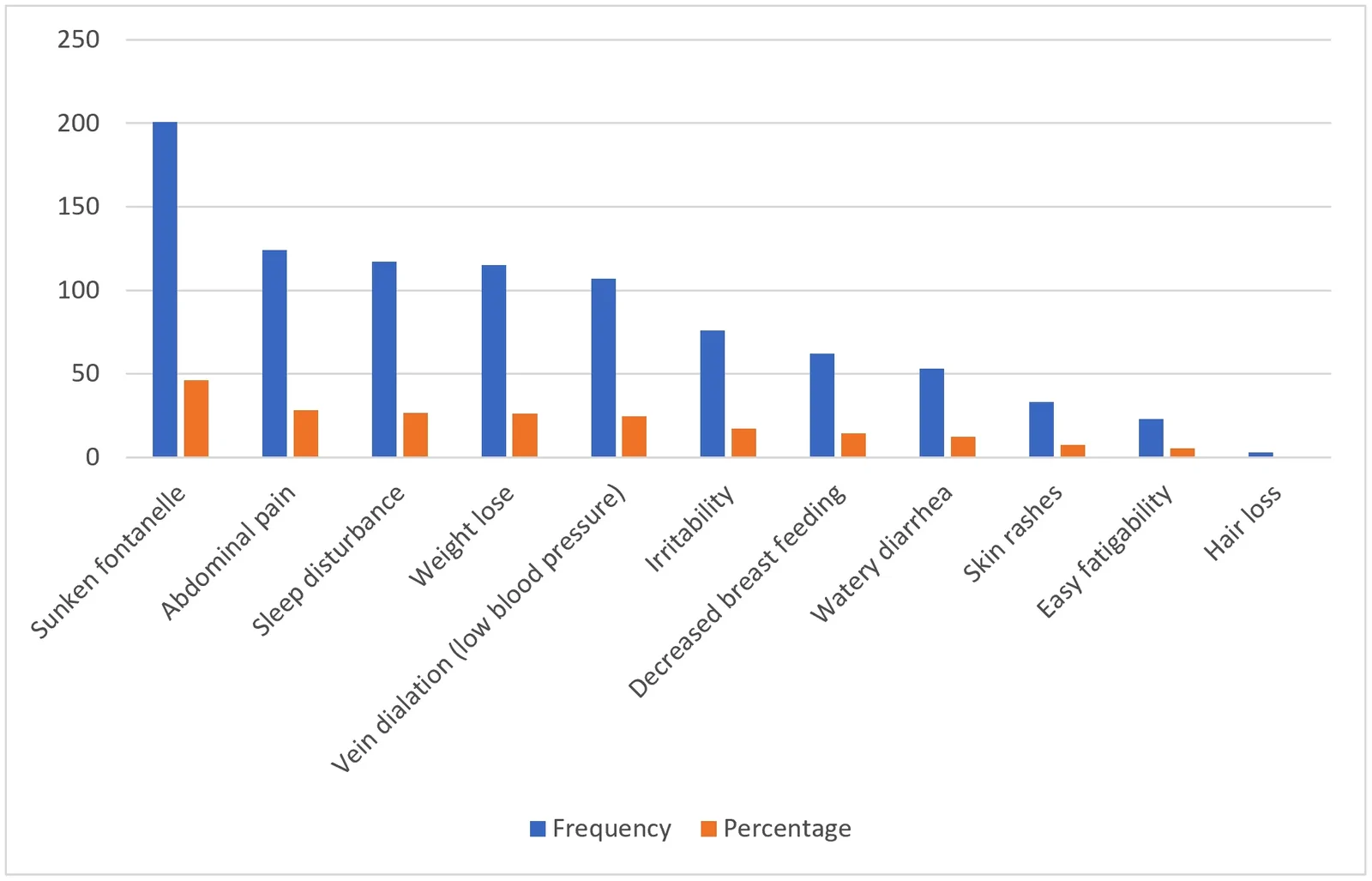
Frequency of common symptom of the severe form of “*feyancho*” (*arusicho*) as described by the community.

### Clinical characteristics of children claimed to have “*feyancho”*

Examinations were conducted by physicians to clinically define the “*feyancho” (arusicho/sidancho)*. It included data on the place of birth, previous episodes, symptom resolution, and gender distribution. The table also covered medical evaluation and hospitalization history, maternal employment, respiratory and environmental exposures, and immunization status. Various symptoms and conditions were detailed, such as fever, sweating, chills, cough, wheezing, shortness of breath, chest pain, runny nose, throat issues, difficulty swallowing, ear pain, red draining eye, muscle ache, joint pain, swollen glands, rash, stiffened neck, seizures, vomiting, diarrhea, bloody urine, yellow skin/eyes, weight loss, irritability, skin rashes, and hair loss, Table 3.

**Table 3:** Clinical characteristics of *“feyancho” (sidancho/ arusicho)* in SNRS, (N = 13), 2023.

Characteristics	Options	Frequency (%)	Characteristics	Options	Frequency (%)
Place of birth	Facility-based	11(84.62)	Muscle ache	Yes	0
				No	8(61.54)
	Home delivery	2(15.38)		UK	5(38.46)
Ever have similar episode	Yes	3(23.08)	Joint pain/ swelling	Yes	0
	No	10(76.92)		No	13(100)
Similar case in the vicinity	Yes	3(23.08)	Enlarged/swollen glands	Yes	1(7.69)
	No	10(76.92)		No	12(92.31)
Symptom-resolved without intervention	Yes	3(23.08)	Rash	Yes	9(69.23)
	No	10(76.92)		No	4(30.77)
Sex	Male	9(69.23)	Red draining eye	Yes	1(7.69)
	Female	4(30.77)		No	12(92.31)
Ever evaluated by physician before	Yes	1(7.69)	Bloody urea	Yes	0
	No	12(92.31)		No	13(100)
Ever hospitalized	Yes	1(7.69)	Yellow skin/eye	Yes	0
	No	12(92.31)		No	13(100)
Mother works outside home	Yes	3(23.08)	T^0^c raised	Yes	0
	No	10(76.92)		No	3(23.08)
Ever have respiratory distress	Yes	0	Wheezing bronchi	UK	10(76.92)
	No	13(100)		Yes	0
Ever exposed to soil	Yes	4(30.77)	Mucosal lesions	No	2(15.38)
	No	9(69.23)		UK	11(84.62)
Have pets in the same house	Yes	5(38.46)	Altered mental status	Yes	2(15.38)
	No	8(61.54)		No	11(84.62)
Ever have contacts with pets	Yes	2(15.38)	Neurological abnormalities	Yes	0
	No	11(84.62)		No	13(100)
Ever have contacts with rodents	Yes	4(30.77)	Hepatosplenomegaly	Yes	0
	No	9(69.23)		No	13(100)
Immunized	Yes	13(100)	Jaundice	Yes	0
	No	0		No	4(30.77)
Ever used medication including OTC	Yes	7(53.85)	Excessive crying	UK	9(69.23)
	No	6(46.15)		Yes	0
Ever have depressed fontanelle	Yes	0	Constipation	No	13(100)
	No	6(46.15)		Yes	9(69.23)
	Unknown	7(53.85)		No	4(30.77)
Current fever	Yes	7(53.85)	Failure to thrive	Yes	1(7.69)
	No	6(46.15)		No	12(92.31)
Current sweet	Yes	10(76.92)	Reduced breast Feeding	Yes	5(38.46)
	No	3(23.08)		No	8(61.54)
Current chills	Yes	2(15.38)	Abdominal pain	Yes	4(30.77)
	No	11(84.62)		No	9(69.23)
Current cough	Yes	7(53.85)	Watery diarrhea	Yes	0
	No	6(46.15)		No	5(38.46)
Current cough with sputum	Yes	0	Weight loss	UK	8(61.54)
	No	13(100)		Yes	10(76.92)
Wheezing	Yes	0	Irritability	No	3(23.08)
	No	12(92.31)		Yes	11(84.62)
	Unknown	1(7.69)		No	2(15.38)
Shortness of breath	Yes	0	Skin rashes	Yes	10(76.92)
	No	12(92.31)		No	3(23.08)
Chest pain	Yes		Hair loss	Yes	10(76.92)
	No	7(53.85)		No	3(23.08)
	Unknown	6(46.15)		Yes	2(15.38)
Runny nose	Yes	0	Stiff neck	No	11(84.62)
	No	13(100)		Yes	1(7.69)
Nose throat	Yes	2(15.38)	Seizures	No	12(92.31)
	No	11(84.62)		Yes	2(15.38)
Difficulty swallowing	Yes	1(7.69)	Vomiting	No	11(84.62)
	No	12(92.31)		Yes	7(53.85)
Ear pain	Yes	1(7.69)	Vomiting with blood	No	6(46.15)
	No	8(61.54)		Yes	0
	Unknown	4(30.77)		No	13(100)

### Clinical manifestations of the “*feyancho*” disease

A total of 12 clinical cases were investigated. The study found that creatinine and serum glutamic-pyruvic transaminase (SGPT) / alanine aminotransferase (ALT) levels were within normal ranges for all participants. However, blood urea levels were elevated in one participant (8.33%). A significant percentage of participants exhibited elevated levels of Serum Glutamic-Oxaloacetic Transaminase (SGOT) aspartate aminotransferase (AST) (58.33%), Alkaline Phosphatase (ALP) (91.66%), and Direct Bilirubin (DB) (58.33%).

Cholesterol levels were within a healthy range across the participants, bilirubin findings (both total and direct) pointed to mild jaundice in some cases. Overall, this study highlights that while most participants exhibit healthy biochemical profiles, a subset presented with abnormal liver enzyme levels and elevated bilirubin, Table 4.

**Table 4:** Clinical chemistry of samples collected from children with Feyancho disease, (N = 12) 2023.

Variable	Range in study	Mean	Range less than 1year	%below normal	%above normal	Laboratory range
Creatinine (mg/dl)	0.21-0.39	0.31	0.2-0.4	0	0	0.9-1.3
Urea (mg/dl)	6.3-18.2	12.21	5–18	0	8.33	16.6-48.5
SGPT U/L	11.3-50.9	24.72	7-56	0	0	0-41
SGOT U/L	5-40	52.69	5-40	0	58.33	0-40
ALP U/L	244.8 −1237.9	701.9	81.9 - 350.3	0	91.66	120-460
TC (mg/dl)	70.9 −147.9	121.92 ± 29.19	<170	0	0	0-200
Glucose (mg/dl)	56.0–108.8	74.83 ± 13.97	60-200	8.33	0	74-109
Albumin (g/l)	3.52-4.66	4.35 ± 38.84	3.4–5.2	0		3.5-5.2
TB (mg/dl)	0.2- 3.28	1.15 ± 1.01	<2.0	0	25	0.2-1.0
DB (mg/dl)	0.23 −4.15	1.40 ± 1.36	<0.3	0	58.33	0-0.2
TP (mg/dl)	6.6 −8.6	7.83 ± 0.58	4.5 −7.0			6.6-8.7

### Hematological parameters of the “*feyancho*” disease

The haematological profile of children with Feyancho disease demonstrated abnormalities across all major blood cell lineages. Mild leukocytosis was observed, characterized by marked lymphocytosis with concurrent neutropenia and increased eosinophil counts, while monocyte and basophil counts were reduced. Red blood cell indices indicated microcytic, hypochromic anaemia, evidenced by reduced RBC count, haemoglobin, haematocrit, MCV, and MCH, with normal MCHC and elevated RDW, suggesting significant anisocytosis. Platelet analysis showed a moderately reduced platelet count with increased PDW, whereas MPV, PCT, and P-LCR remained within normal limits, indicating altered platelet morphology despite preserved platelet size indices. Overall, the findings suggest that Feyancho disease is associated with abnormalities in erythrocyte, leukocyte, and platelet parameters, Table 5.

**Table 5:** Investigation of the hematological parameters of the samples collected from children with Feyancho disease, (N = 12), 2023.

Variable	Min value	Max value	Observed Mean	Normal Mean	Difference	% Difference	Deviation
WBC (×10³/µL)	4.560	18.21	8.84	7.5	+1.34	+17.87%	Elevated
NEU (×10³/µL)	1.300	6.86	3.37	4.75	-1.38	-29.05%	Decreased
LYM (×10³/µL)	2.760	7.33	4.56	2.0	+2.56	+128.00%	Elevated
MON (×10³/µL)	0.200	0.93	0.42	0.6	-0.18	-30.00%	Decreased
EOS (×10³/µL)	0.050	3.24	0.46	0.26	+0.20	+76.92%	Elevated
BAS (×10³/µL)	0.020	0.02	0.02	0.1	-0.08	-80.00%	Decreased
NEU %	21.400	50.80	37.93	60.0	-22.07	-36.78%	Decreased
LYM %	36.000	70.60	53.88	30.0	+23.88	+79.60%	Elevated
MON %	2.400	7.00	4.78	5.0	-0.22	-4.40%	Normal
EOS %	0.700	17.80	3.52	3.5	+0.02	+0.57%	Normal
BAS %	0.100	0.50	0.29	0.5	-0.21	-42.00%	Decreased
RBC (×10⁶/µL)	2.510	4.98	4.12	4.75	-0.63	-13.26%	Decreased
HGB (g/dL)	4.600	14.00	10.07	14.0	-3.93	-28.07%	Decreased
HCT (%)	15.400	40.60	29.67	42.0	-12.33	-29.36%	Decreased
MCV (fL)	54.500	81.80	71.34	90.0	-18.66	-20.73%	Decreased
MCH (pg)	16.200	28.20	24.24	29.5	-5.26	-17.83%	Decreased
MCHC (g/dL)	29.800	37.20	33.86	34.0	-0.14	-0.41%	Normal
RDW-CV (%)	13.300	25.90	17.71	13.0	+4.71	+36.23%	Elevated
RDW-SD (fL)	39.700	55.70	47.12	42.5	+4.62	+10.87%	Elevated
PLT (×10³/µL)	17.000	501.00	223.0	300	-77.0	-25.67%	Decreased
MPV (fL)	7.100	12.40	9.34	9.5	-0.16	-1.68%	Normal
PDW (fL)	14.300	16.40	15.61	11.5	+4.11	+35.74%	Elevated
PCT (%)	0.120	4.55	0.214	0.23	-0.016	-6.96%	Normal
PLCR	0.172	0.44	0.25	0.28	-0.03	-10.71%	Normal

## Discussion

Illnesses in newborns typically range from mild skin rashes and common colds to more severe infections and genetic defects. While some are easily managed at home, others may require prompt medical attention. “*Feyancho*” among Sidama nation is characterized symptomatically and almost exclusively managed at home. Yet, its western equivalence has not yet been established. To establish the etiological manifestations, communities’ description of the diseases, signs and symptoms, clinical samples were analyzed. This study, the first in its kind, has found out that the overall prevalence of “*feyancho*” was 59.52%. The disease is higher among females (63.93%) than males (55.48%). Clinical evaluations of “*feyancho*” cases revealed the possibility of **microcytic anemia, high lymphocytes, low neutrophils, increased** RDW (Red Cell Distribution Width)**, and anisocytosis, suggest iron deficiency, inflammation, or chronic infection.**

Slightly higher prevalence among female might indicate that females are either more susceptible to “*feyancho*” disease possibly due to biological or environmental factors. A previous study conducted in the region found that all children tested were positive for fungal elements (dermatophytosis) in microscopy with 97.6% exhibited growth in culture [7]. The skin manifestations observed in the current study participants may agree with the report.

Regarding disease classification, participants categorized “*feyancho*” into two types: *sidancho* (a milder form) and *arusicho* (a severe form). *Arusicho* is associated with more severe clinical manifestations mainly manifested as sunken fontanelles and ultimately leads to death if left untreated with traditional treatments.

A sunken fontanel can be caused by dehydration, kwashiorkor (severe protein deficiency), failure to thrive (poor growth in children), and serious medical conditions like toxic megacolon and diabetes insipidus. The understanding of this condition as well as prevention and treatment have corresponded to the specific cultural setting and is a culturally interpreted symptom [8]. The study of Tswana-speaking urbanites’ cultural perceptions of diarrhoeal disease in infants is often linked to sorcery, with concepts like “phogwane” (sunken fontanelle) and “kokwana” (intestinal snake) used to explain its origins [9]. This concept of a sunken fontanelle overrides among several communities and is attributed to various understandings and treatment aspects, and culturally informed and plausible approaches are important to such conditions [10].

A significant majority (66.51%) of respondents believed that mothers affected by “*feyancho*” increased the likelihood of their children contracting the disease. Maternal-to-child transmission was considered the primary route (57.34%), followed by transmission through breastfeeding (40.37%) and direct contact among children (14.45%). Genetic and airborne transmissions were believed to play a minimal role. This indicates that cultural beliefs about transmission heavily influence local health perceptions and may impact preventive health behaviors.

Medical evaluations provided additional insights into the disease’s presentation. Notably, symptoms such as excessive crying, weight loss, and irritability were commonly observed in affected children. Despite the widespread community belief in maternal transmission, clinical examinations did not find strong correlations with maternal health conditions. Furthermore, hospitalization rates among affected children were low, suggesting either underreporting of severe cases or limited access to healthcare facilities.

Laboratory analysis revealed that a significant portion of participants exhibited elevated SGOT, ALP, and direct bilirubin (DB) levels, suggesting potential liver dysfunction or stress, possibly linked to the underlying conditions affecting liver function or even possible toxicity of herbal medicine use. In contrast, creatinine and urea levels remained mostly within normal ranges, indicating no major kidney issues. However, the slight elevation in urea observed in some participants warrants further assessment of hydration status or early signs of kidney function impairment. While cholesterol levels were within a healthy range across participants, bilirubin findings (both total and direct) pointed to mild jaundice in some cases—an expected condition in infants but one that requires close monitoring. Overall, this study highlights that while most participants exhibit healthy biochemical profiles, a subset presents with abnormal liver enzyme levels and elevated bilirubin, warranting further investigation to rule out any underlying health concerns.

The haematological analysis revealed several key findings. Red blood cell (RBC) parameters suggest a prevalence of anaemia among participants, as evidenced by low RBC count, haemoglobin, and haematocrit levels. The reduced mean corpuscular volume (MCV) and mean corpuscular haemoglobin (MCH) indicate microcytic anaemia, commonly associated with iron deficiency [11,12]. Despite these findings, the mean corpuscular haemoglobin concentration (MCHC) remained within normal limits, suggesting that while red blood cells were smaller; their haemoglobin concentration was not significantly altered. An elevated red cell distribution width (RDW) further supports the presence of anisocytosis, a condition characterized by varying RBC sizes, often seen in nutritional deficiencies [13].

Platelet counts were within the normal range, indicating no major clotting disorders, though platelet distribution width (PDW) were slightly reduced, suggesting variations in platelet size and potential changes in platelet production. Additionally, elevated lymphocyte levels may point to underlying viral infections or chronic inflammatory conditions [14]. Despite slight increases in PDW, platelet function appears normal, as overall platelet counts remain within the expected range. Lastly, eosinophil levels show mild elevations in some cases, which could indicate allergic reactions or parasitic infections among certain participants [15]. The lower-than-normal neutrophil percentage likely correlates with the relatively higher lymphocyte count, reinforcing the possibility of underlying viral infections.

Overall, these hematological abnormalities suggest that children with Feyancho disease experience multisystem hematological disturbances, dominated by microcytic iron-deficiency anemia, lymphocyte-predominant leukocyte responses, relative neutropenia, eosinophilia, and anisocytosis.

## Conclusion

This study provided valuable insights into the characteristics and health conditions associated with “*feyancho*”. The findings underscore the need for targeted interventions to address both healthcare access and community health education to mitigate the impact of “*feyancho*”. Given the high prevalence of “*feyancho*” and the strong cultural beliefs surrounding its transmission, community-based health education programs should be implemented. These programs should focus on improving awareness of evidence-based disease transmission and preventive measures. Currently, cases are managed through traditional methods. Strengthening collaboration between healthcare providers and local communities will be very crucial in improving child health outcomes in the region. Further epidemiological studies that integrate medical diagnostics with community perceptions could help bridge the knowledge gap and inform public health interventions. The findings also support the need for comprehensive clinical evaluation, including assessment of iron status (serum ferritin, transferrin saturation), inflammatory markers (CRP), stool examination for intestinal parasites, and evaluation for concurrent viral infections.

## List of abbreviations:

ALP: Alkaline Phosphatase
ALT: Alanine Aminotransferase
AST: Aspartate Aminotransferase
CAM: Complementary and Alternative Medicine
DB: Direct Bilirubin
EOS: Eosinophils
FGD: Focus Group Discussion
GPS: Global Positioning System
HCT: Hematocrit
HGB: Hemoglobin
IKS: Indigenous Knowledge Systems
LYM: Lymphocytes
MCH: Mean Corpuscular Hemoglobin
MCHC: Mean Corpuscular Hemoglobin Concentration
MCV: Mean Corpuscular Volume
MPV: Mean Platelet Volume
NEU: Neutrophils
OTC: Over the Counter
PCT: Plateletcrit
PDW: Platelet Distribution Width
PhD: Doctor of Philosophy
PIM: Pediatric Integrative Medicine
PLCR: Platelet Large Cell Ratio
PLT: Platelets
RDW-CV: Red Cell Distribution Width-Coefficient of Variation
RDW-SD: Red Cell Distribution Width-Standard Deviation
SGOT: Serum Glutamic-Oxaloacetic Transaminase
SNRS: Sidama National Regional State
SPGT: Serum Glutamic-Pyruvic Transaminase
TB: Total Bilirubin
TC: Total Cholesterol
TP: Total Protein
WBC: White Blood Cells

## Supporting information

S1 File Prevalence and Characterization of Infant Health Conditions Treated by the Ameessa Plants among Sidaama People and its Ethnobotanical Survey, Nutritional Status, Bioactivity and Safety. (DOCX)
