# Supplementary material for: Traditional knowledge of childhood illnesses among the Sidama nation, clinical presentation and hematological findings
Source: PLoS One. 2026 Nov 10;21(11):e0340246. doi: 10.1371/journal.pone.0340246 (PMC13750600; doi:10.1371/journal.pone.0340246)
Supplement: Supplementary file 1 [file pone.0340246.s001.docx]

Prevalence and Characterization of Infant Health Conditions Treated by the Ameessa Plants among Sidaama People and its Ethnobotanical Survey, Nutritional Status, Bioactivity and Safety

Serawit Deyno, B. Pharm, M.Sc, PhD, School of Pharmacy, Hawassa University, Hawassa, SNRS

Tsadiku Getachew, MD, Adaare General Hospital, Hawassa, SNRS

Maeregu Daniel, BSc in Chemical Engineering, Institute of Technology, Hawassa University, Hawassa, SNRS

Sintayehu Fekadu, B. Sc, M.Sc, PhD, School of medical laboratory technology, Hawassa University, Hawassa, SNRS

Menfes Tadesse, PHD, School of Natural Resources and Environmental Studies, Wondo Genet College of Forestry and Natural Resources, Hawassa University

Nigatu Tuasha, PhD in Biomedical Sciences, Hawassa College of Teachers Education, Hawassa, SNRS

*January, 2022*

*Hawassa*

Contents

[Executive summary	5](#_Toc96031009)

[1.	Introduction	7](#_Toc96031010)

[1.1.	Background	7](#_Toc96031011)

[1.2.	Statement of the Problem	8](#_Toc96031012)

[1.3.	Significance of Research	9](#_Toc96031013)

[1.4.	Research question	9](#_Toc96031014)

[1.5.	Objectives of the study	9](#_Toc96031015)

[2.	Literature review	10](#_Toc96031016)

[2.1.	Diversity of Medicinal Plants and their Role in the Health System of Ethiopia	10](#_Toc96031017)

[3.	Methods and materials	12](#_Toc96031018)

[3.1.	Study area	12](#_Toc96031019)

[3.2.	Study design	12](#_Toc96031020)

[4.	Description of the action and its effectiveness	13](#_Toc96031021)

[5.	Expected output	13](#_Toc96031022)

[6.	Beneficiaries	14](#_Toc96031023)

[7.	Ethical Clearance	14](#_Toc96031024)

[8.	Monitoring and Evaluation	15](#_Toc96031025)

[9. General breakdown of the costs and costs by phases of each year	15](#_Toc96031026)

[References	15](#_Toc96031027)

[Theme One: Ethnobotany survey and Prevalence of ameessa plants use and Clinical Characterization of Feyancho Disease among Sidaama people	17](#_Toc96031028)

[Executive Summary	18](#_Toc96031029)

[1. Introduction	19](#_Toc96031030)

[1.1.	Background	19](#_Toc96031031)

[1.3 Conceptual Framework	20](#_Toc96031032)

[2. Significance of the Study, Objectives, Statement of the Problem and Research Questions	21](#_Toc96031033)

[2.1 Significance of the Study	21](#_Toc96031034)

[2.2 Objectives	22](#_Toc96031035)

[2.2.1 General Objective	22](#_Toc96031036)

[2.2.2 Specific Objectives	22](#_Toc96031037)

[2.3 Statement of the problem and Research Questions	22](#_Toc96031038)

[3. Materials and Methods	23](#_Toc96031039)

[3.1. Materials	23](#_Toc96031040)

[3.2. Study design	23](#_Toc96031041)

[3.3. Sample collection Area: the Community	23](#_Toc96031042)

[3.4. Reconnaissance survey	25](#_Toc96031043)

[3.5. Informant selection	25](#_Toc96031044)

[3.6. Ethnobotanical Data Collection	25](#_Toc96031045)

[3.7. Floristic Data Collection and Identification of Plant Species	26](#_Toc96031046)

[3.8. Determination of the prevalence of infantile illness	26](#_Toc96031047)

[3.9. Eligible criteria of the participants in a case study	27](#_Toc96031048)

[3.10. Data collection	27](#_Toc96031049)

[3.11. Data Analysis	27](#_Toc96031050)

[4. Description of the action and its effectiveness	29](#_Toc96031051)

[5. Expected output	30](#_Toc96031052)

[6. Beneficiaries	30](#_Toc96031053)

[7. Ethical Clearance	30](#_Toc96031054)

[8. Monitoring and Evaluation	31](#_Toc96031055)

[9. Work Plan	1](#_Toc96031056)

[10. Cost of the Study and Budget Breakdown	0](#_Toc96031057)

[References	3](#_Toc96031058)

[THEME TWO: BIOACTIVITIES OF SELECTED AMEESSA PLANTS AND DETERMINATION OF NUTRITIONAL VALUES	7](#_Toc96031059)

[1.	Introduction	8](#_Toc96031060)

[1.1.	Background	8](#_Toc96031061)

[1.2.	Objective of the study	9](#_Toc96031062)

[1.2.1.	Specific objectives	9](#_Toc96031063)

[1.3.	Significance of the study	9](#_Toc96031064)

[2.	Methods and materials	9](#_Toc96031065)

[2.1. Plant extraction	9](#_Toc96031066)

[2.2. Preliminary Phytochemical Screening Tests	10](#_Toc96031067)

[2.3. Isolation by Chromatographic Techniques	10](#_Toc96031068)

[2.4. Melting point determination of isolated compound	11](#_Toc96031069)

[2.5. Spectroscopic analysis	11](#_Toc96031070)

[2.6. Antibacterial Activity determination	12](#_Toc96031071)

[2.6.1.	Preparation and Dilution of Test Solutions	12](#_Toc96031072)

[2.6.2.	Bacterial Inoculum Preparation	12](#_Toc96031073)

[2.6.3.	Minimum Inhibitory Concentration	12](#_Toc96031074)

[2.7.	Antifungal activity	13](#_Toc96031075)

[2.7.1.	Inoculum preparation	13](#_Toc96031076)

[2.7.2.	Broth microdilution	13](#_Toc96031077)

[2.9.	Data management	14](#_Toc96031078)

[3.	Description of the action and its effectiveness	14](#_Toc96031079)

[4.	Expected output	15](#_Toc96031080)

[5.	Beneficiaries	15](#_Toc96031081)

[6.	Ethical Clearance	15](#_Toc96031082)

[7.	Monitoring and Evaluation	15](#_Toc96031083)

[8.	Work plan	16](#_Toc96031084)

[9.	General breakdown of the costs and costs by phases of each year	17](#_Toc96031085)

[THEME THREE: ACUTE AND SUB-ACUTE TOXICITY STUDIES OF THE EXTRACTS OF SELECTED AMEESSA MEDICINAL PLANTS IN ANIMAL MODELS	21](#_Toc96031086)

[1.	Introduction	23](#_Toc96031087)

[4.	Description of the action and its effectiveness	26](#_Toc96031088)

[5.	Expected output	26](#_Toc96031089)

[6.	Beneficiaries	26](#_Toc96031090)

[7.	Ethical Clearance	27](#_Toc96031091)

[8.	Monitoring and Evaluation	27](#_Toc96031092)

[9.	General breakdown of the costs and costs by phases of each year	27](#_Toc96031093)

[10.	Work Plan	28](#_Toc96031094)

Main Theme

Prevalence and Characterization of Infant Health Conditions Treated by the Ameessa Plants among Sidaama People and its Ethnobotanical Survey, Nutritional Status, Bioactivity and Safety

# Executive summary

**Background:** Indigenous knowledge has been helping scientist to guide their work where researchers drop into communities, gather data and analyze the findings. Ethnobotany, study of the biological knowledge of particular ethnic groups, focuses on how plants are used, managed and perceived by a society and includes plants used for food, medicinal, rituals, social life and other purposes. There is no well documented data on the ethnobotany of ameessa plants in Sidaama. The present study aims to investigate the ethnobotanical knowledge of Sidaama people about the ameessa plants and the health conditions of the infants treated. Bioassays to determine the effectiveness against various pathogens, nutritional status and safety profiles of the selected ameessa plants will also be evaluated.

**Method:** Ethnobotanical survey will be conducted through reconnaissance survey and identification of informants from the sampling sites. Floristic data will be collected, numbered and pressed carefully for identification of the plant species using authorized herbarium settings. Ethnobotanical data will be collected mainly by interview and observation-based field study. The Data collection will be performed under close monitoring of the supervisors. STATA will be used for data analysis. The prevalence of the illness of infants treated by the ameessa plants will be determined. Characterization the diseases will be conducted by taking signs and symptoms, clinical chemistry, hematology and microbiological culturing.

The efficacy of Ameessa plant will be evaluated in the hypothesized disease model during clinical characterization study. The prioritized ameessa plants will be extracted and isolation of its bioactive components will be carried out. The antibacterial activity, antifungal activity, anti-parasitic and anti-helminthic activity will be determined as per standard guidelines. Safety study will be conducted using animal models. Nutritional content analysis will also be determined. Data entry, validation, data quality assurance approaches and appropriate statistical techniques will be done. Statistics analysis will be carried out using relevant models.

This project will be accomplished in three years, from April 2022- June 2025. The estimated total budget required for the successful accomplishment of this project is about 2,636,084 (Two million six hundred thirty six thousand and eighty four).The findings will be presented in conference, public lectures and publication of journal articles. Policy recommendation will be made to governments. The study is expected to enable to pass indigenous knowledge, practices, skills and wisdom of Sidaama people from generation to generation in a well-documented manner supported by empirical evidences. Optimization of the traditional treatment and development of modern medicine from ameessa is expected.

# 1. Introduction

## 1.1. Background

Indigenous knowledge refers to the accumulation of knowledge, rule, standards, skills, and mental set, which are possessed by local people in a particular area. Ethnobotany is one of the areas where indigenous practice continues to be a source for knowledge about medicines, crops, agricultural techniques, conservation and management, and so much more. Human beings have used plants, plant constituents, herbal preparations, and finished herbal products for centuries, among others as foods; as medicinal agents; as stimulants, narcotics, and hallucinogens; for clothing and shelter; as aromatics, cosmetics, and dyes; and to prepare poisonous arrow and spearheads for warfare and hunting. Most indigenous knowledge has had only a little success attracting scientific researches. Incorporating indigenous knowledge into science can create rapid growths in understanding and revolutionizing human benefits (Sidik 2022).

In Ethiopia, long history of using traditional medicinal plants for combating various ailments were documented in the collection of medico-religious manuscripts of the Axumite kingdom (Pankhurst, R., 1990). It is asserted that about 80% of the Ethiopian people depend on traditional medicine for their health care and more than 95% of traditional medicinal preparations in Ethiopia are sourced from plants (Kassaye *et al.,* 2006),. Since each ethnic group or a community has its own concepts of health or illness and therefore information on how the plant is used by an ethnic group is extremely important, it is crucial to investigate and document the ethnobotanical aspects of different community with myriad traditional medicine practices. Recently, ethnobotanical studies are gaining due attention globally and reasonably acceptable proportion of studies are being conducted in different parts of Ethiopia. In Sidaama, very few ethnobotanical studies were conducted and reported the use of traditional medicinal plants for the treatment of various health conditions (Kewessa *et al.,* 2015, Asnake *et al.,* 2016, Tefera & Kim 2019,).

Of various health conditions of infants called “lagote dhibba”. The condition is described with different names in different areas in Sidaama National Regional State (SNRS). For instance, feyancho, sidaancho or arusicho are some of its names among others. The descriptions of the health condition are very close to one another while the medicinal plants traditionally used for the treatment of the condition vary dramatically. Scrutiny of the application is also a very cautious one. Though there exist sporadic reports of the presence of the knowledge among different Sidaama community, there is no well documented data on the ethnobotany of ameessa in Sidaama. The present study aims to investigate the ethnobotanical knowledge of Sidaama people about the ameessa plant and the health conditions of the infants treated.

The first theme of this thematic research will be ethnobotanical survey to document the indigenous knowledge and practice on the use of Ameessa plant for the treatment of infantile health condition called fayancho. In this survey reconnaissance survey and identification of informants from the sampling sites will be done first. Ethnobotanical data will be collected mainly by interview and observation-based field study. Floristic data will be collected, numbered and pressed carefully for identification of the plant species. Careful recording of ecological data including altitude and geographical coordinates will be done using GPS at the spot during specimen collection. The prevalence of the “feyancho/lagote dhiba” is not described in the region and thus the study will also determine the prevalence of the infantile illness treated by ameessa plants. Data entry validation, data quality assurance approaches and appropriate statistical techniques will be done.

In the second theme, characterization of the diseases condition will be conducted. Clinical samples will be collected from the assenting/consenting participants/parents and diagnosis will be conducted to identify is the etiologic agent of the infantile illnesses. Physical diagnosis, sign and symptoms and laboratory diagnosis will be conducted to define the possible disease conditions.

In the third theme, the effectiveness of ameessa plants for possible health condition in infants will be evaluated. If the conduct during theme two indicates possibility of infection, plant extracts will be evaluated for anti-microbial, anti-fungal, anti-helminthic and anti-parasitic activities. Nutritional status of the ameessa plants will also be studied.

In the fourth theme, the safety of the ameessa plants will be evaluated in animal models.

## 1.2. Statement of the Problem

Plants have proved promising healing effects to various disease conditions and even contributed to the pool of modern medicine. Ameessa has long been used for the treatment of various health conditions in infants. They are long been used for the treatment of infantile health condition called feyancho. But these disease conditions are not clearly defined and the prevalence of the disease is not yet determined. The common plants used for these conditions are also not properly documented. Furthermore, the safety and effectiveness of the plants used for the management of the stated infantile condition were not yet evaluated. The nutritional status of ameessa plants is not experimented either.

## 1.3. Significance of Research

Indigenous knowledge has contributed to a greater extent to the development of modern medicine. Though medicinal plants are commonly used in Ethiopia, their efficacy is poorly studied. Investigation of their effectiveness will help to optimize traditional usage and facilitate modern product development. There is a continuing need to query the indigenous peoples’ knowledge and practice of various diseases conditions. These studies primarily focus on identification of commonly used ameessa plants ethnobotanical knowledge, prevalence of the infantile disease treated by the ameessa plants, clinical characterization of the disease treated by the ameessa plants, nutritional value investigation, various bioactivities and safety evaluations. Therefore, the project will have significant scientific and community based implications.

## 1.4. Research question

This study focuses on evaluating ethnobotanical uses of ameessa plants used by the Sidaama people for the management of infantile health conditions and investigates the bioactivities and determines its nutritional values. Besides, the prevalence of infantile health conditions treated by the ameessa will be determined. Clinical characterization of its etiologic agents will be done and the safety of the ameessa will be studied in animal models. To this end, the following research questions will be addressed.

1. How diverse are the medicinal plants called ameessa plants? What are the ethnobatanical knowledge associated with the ameessa plants?
2. What are the infantile illnesses called feyaancho ( sidancho/arusicho) and how are these diseases characterized clinically? How prevalent are these infantile illnesses?
3. How effective are the ameessa plants for the management of infantile health conditions? Are the ameessa plants endowed with anti-microbial. antifungal, anti-helminthic or anti-parasitic activities?
4. Does it have a nutritional value and enhance brain development?
5. How safe are ameessa plants?

## 1.5. Objectives of the study

**Main objective**

- To carry out an ethnobotanical survey, evaluate ethnopharmacology, phytochemistry and pharmacology of ameessa medicinal plants used in Sidaama for the management of infantile diseases, determine the prevalence of infantile illness treated by ameessa, clinically characterize the etiology, conduct selected bioassays and test the safety of the ameessa medicinal plants.

**Specific objective**

- To document ethnobotanical evidence on medicinal plants used as the ameessa for the treatment of infantile health conditions by Sidaama people
- To determine the clinical characteristics of disease conditions locally known as feyancho ( arusicho/sidancho
- To describe the prevalence of infantile illness treated by ameessa in the Sidaama national Regional State
- To evaluate the bioactivities (antimicrobial and antifungal) of ameessa plants and assess its nutritional values
- To evaluate the safety of ameessa plants using animal models

# 2. Literature review

## 2.1. Diversity of Medicinal Plants and their Role in the Health System of Ethiopia

Pankhurst (1990) indicated that the antiquity of the traditional use of medicinal plants in Ethiopia could not be simply over looked. The collection of medico-religious manuscripts of the Axumite kingdom confirms the long history of using traditional medicinal plants for combating various ailments in Ethiopia (Pankhurst, 2001). It has been noted that testimony to this is found in medical textbooks that have been written in Ge’ez, or even Arabic which were written between the mid of 17^th^ and beginning of the 18^th^ century (Gebre Egziaber *et al., 1979*; Debela *et al., 1999*).

The increasing richness of plants toward the equator is related primarily to climatic factors, such as water availability (Mutke *et al.,* 2001); however, the diversity of land variations such as topographic, is also important. Ethiopia’s boundaries encompass the major part of the eastern African highland massif. On the northern and western boundaries lie the foothills of the main massif. The Great Rift Valley cuts diagonally across the country from north east to south, creating a vast depression. The dry areas have isolated the highlands. Thus, there is great variation of altitude from 116 meters below sea level to 4620 meters above sea level. Rainfall also varies widely in amount and distribution. These factors strongly influence Ethiopia’s extraordinary range of terrestrial and aquatic ecosystems, and have contributed to a high diversity and rate of endemism (Awas *et al.,* 2003). The existence of such diverse ecosystems has endowed Ethiopia with diverse vegetation types. Ethiopia is a tropical country with varied macro and micro-climatic conditions that have contributed to the formation of diverse ecosystems inhabited with a great diversity of life forms of both animals and plants. These plants vary in their distribution over the country due to the varied geographic and climatic conditions (Getahun, 1976). The flora of Ethiopia is diverse with about 6,500-7,000 species of higher plants with about 12% endemic elements (Gebre Egziaber, 1991; UNEP, 1995). Among African countries, Ethiopia is often quoted as one of the six countries of the world where about 60% of the plants are said to be indigenous with their healing potential (Desta, 1984).

The diversity is also considerable in the lower plants but exact estimate of these have to be made. The genetic diversity contained in the various biotic make up is also high thus making the country a critical diversity hot spot for plants (Bekele, 2007). Jansen asserts that Ethiopia has rich medicinal plant lore and points out that almost all plants of the Ethiopian flora are used somewhere somehow medicinally (Jansen, 1981).

Eighty percent (80%) of the Ethiopian people depend on traditional medicine for their health care, and more than 95% of traditional medicinal preparations in Ethiopia are made from plant origin. There are about 887 medicinal plant species that are currently used by the Ethiopian people (<http://www.etflora.net>); some argue there are over 1,000 medicinal plant, however. The majority of the medicinal plants are herbs (271 species), followed by shrubs (168 species) and trees (110 species). According to recent reports, endemic medicinal plants of Ethiopia are over 33 species (Tena Regassa *et al.,* 2014). Most of the medicinal plant species are found in the wild (357 (40.2%) species) and therefore, the threats and trends are similar to those of the forest plant species (<http://www.etflora.net>).

Recent ethnobotanical study in Gorichei District has documented about 75 plant species of which, 55 are found to be used to treat human diseases, 10 species used as veterinary medicine and 10 species are used for both human and livestock ailment management (Misrak Bekele *et al.,* *2014)*. The other ethnobotanical study in Wondo Genet District of Sidaama zone has documented the use of 85 plant species used to treat 61 ailments of which 54 medicinal plants are used to treat human ailments, 8 ethno-veterinary and 23 for both human and livestock ailments (Sintayehu Tamene *et al.,* 2011)*.* Regassa, (2013) reported forty one (41) commonly used indigenous medicinal plants used to treat 32 human ailments from the collections of Hawassa College of Teacher Education forest, which have been collected and cultivated form different parts of Sidaama. Tuasha *et al.,* (2018) reported 71 medicinal plants traditionally used for the treatments of various forms of malignancies and other human ailments and among them are reported some ameessa plants.

Each ethnic group has its own concepts of health or illness and therefore information on how the plant is used by an ethnic group is extremely important (Elisabetsky and Posey, 1986 as in Rates, 2001). The signs and symptoms should be translated, interpreted and related to western biomedical concepts, thus allowing a focused study of a particular therapeutic property (Rates, 2001). Ethnobotanical studies are useful in documenting, analyzing and disseminating of knowledge and interaction between biodiversity and human society, how diversity in nature is used and influenced by human activities (Martin, 1995).

# 3. Methods and materials

## 3.1. Study area

Sidaama regional state is bordered on the south by the Oromiya National Regional State and Gedeo zone, on the west by the Wolayita zone, and on the north and east by the Oromiya National Regional State. Sidaama has geographic coordinates of latitude, north: 5′ 45″ and 6′ 45″ and longitude, east, 38′ and 39′. It has a total area of 10,000 km2. Sidaama accounts for 4.0% of the National population of about 115 million (CSA, 2019).). Hawassa, capital of Sidaama National Regional State is located at an altitude ranging from 1708 meters above sea level, 7^o^3’N38^o^28’E latitude and longitude and is found in the great rift valley belt at the shore of Lake Hawassa, which has a size of a 129 square kilometers.

## 3.2. Study design

This study comprises field based survey to clinical data collection and laboratory-based experimental study. Ethnobotanical survey will be conducted with structured questionnaire and focused group discussion. The prevalence of the infantile health condition will be determined by taking households as sampling units where each birth is considered for the determination of the prevalence. All infants, whose parents/guardians sign consent, with signs and symptoms of the disease and that fulfilled eligibility criteria will be enrolled into the study from the community with the assistance of Health Extension Workers other trained healthcare workers. The participants grouped as having the disease condition will be examined by physician and proper samples will be collected for the clinical studies. Following, the disease will be characterized. During the third phase, selected ameessa plants will be extracted and tested for their bioactivities against pathogens (bacteria, fungi, protozoa or helminthiases) taking into account the findings of thematic two. Nutritional analysis of the plants will also be investigated during this time. In the fourth phase, the safety of promising plants will be evaluated using appropriate animal models.

# 4. Description of the action and its effectiveness

The main actions to be undertaken are mobilizing resources to collect ethnobotanical and floristic data, document the priceless traditional ameessa medicinal plants knowledge of the community before it is decimated, document the definitions/characteristics of the health conditions of infants treated by ameessa and identify priority ameessa MPs for further in-depth bioassays using ethnobotanical tools. The prevalence of the infantile health condition treated by various ameessa plants will be determined. Phytochemical analysis will also take place and the extracts will be tested for their bioactivities. Compound isolation will be guided by the bioactivities. Nutritional value of the ameessa plants will also be investigated to test the hypothesis that physical conditions and brain development of the infants improve following treating the infantile health conditions by ameessa. Though, there is wider use of different plants as ameessa, there is no safety profile for each of those plants. Therefore, we will investigate acute and sub-acute toxicities of the selected ameessa plants. The sought results will be achieved if all stakeholders act responsibly and timely. The role of the funder is crucial in that the planned activities will be executed only when the funds are released. Therefore, the funder is a limiting factor for the effective implementation of the project.

# 5. Expected output

A majority of the traditional knowledge maintained by transferring from one generation to the other through oral dictation is at the verge of extinction as it is covered up by strict secrecy. Most of the time, the age of elderly parents come to an end before transferring the knowledge the entrusted older son. Though some TMPs knowledge seems to be in public domain, it should not be taken for granted that it vanishes in time as population ages. Therefore, documenting the TMPs knowledge properly is the only reliable way to salvage the knowledge. The endpoint of this ethnobotanical survey (theme one) will be publishing the finding in the reputable international journal and carry on the bioassays with the prioritized ameessa plants.

Following the documentation of the ethnobotanical knowledge of the ameessa plants, prevalence of the infantile health condition treated by ameessa plants will be determined and the clinical characterization of the etiology will be investigated. Experimental study will be followed where bioactivity of the ameessa plants are investigated. The selected bioassays (anti-microbial, anti-fungal, anti-protozoal, anti-helminthic etc.) will be carried out and the findings will be published in international reputable journals. Nutritional values of the ameessa plants will be investigated and documented properly. This will also be communicated to the scientific community properly. The withstanding issue with traditional medicinal plant usage is the question of its safety. Here, we will study the safety of the most widely used ameessa plants and generate reliable data on acute and sub-acute toxicities. Of note, the finding will be communicated to the wider community through various means of communication. The ultimate goal will be salvaging the traditional knowledge, generate data on bioactivities, the nutritional value and safety profiles. Here, the knowledge generated will be practically used by the rural and urban residents to manage similar health conditions in infants, now supported with experimental evidence. Potentially, there will be at least six (6) reputable publications to be published in prestigious international journals. If patentable formulations and/or active compounds are isolated, its fate will be decided very wisely following consented agreement with the funder and other concerned stakeholders.

# 6. Beneficiaries

Nowadays, ameessa plant is not only used by a rural community. It is widely practiced in urban settings. Therefore, a proper documentation of the medicinal plants used as ameessa benefits rural and urban community of the SNRS. In addition, in a wider context, the finding benefits the humankind in various ways. Various data generated on ameessa plant benefits the scientific community as well us wider users. Scientists may plan further in-depth scientific investigation on any of the ameessa aspects. A wider community may choose a better ameessa plant based on empirical evidence generated through scientific investigation. Besides, the issue related with safety will be alleviated and the wider community can utilize the ameessa plants without unforeseen threat of the unknowns. The rural community whose traditional knowledge is documented will duly be acknowledged. Reasonable share of the benefit will be considered based on the international guidelines on the same (Friso, et *al.* 2020).

# 7. Ethical Clearance

Ethical clearance will be obtained from the Ethical Review Committee of Hawassa University. Permission will be obtained from the Sidaama National Regional State, and concerned woreda and kebele administrators. Written or informed consent/assent will be obtained from study participants after a brief explanation about the objectives of the study to the participants. Confidentiality will be maintained during and after the study period and private data will never be disclosed for any third party, fully or partially.

# 8.  Monitoring and Evaluation

Internally, the researchers will carry out the project with strict research ethics and will disclose relevant information about the conduct to the concerned quality assuring body upon reasonable request. The team will evaluate the progress of the project periodically and will document the minute of the meeting. External authority, delegates of the research and technology transfer office, may convene a relevant monitoring and evaluation meetings. The progress report and final report of the project, upon completion, will be duly communicated as per scheduled by the office. Finally, the publication will be produced following proper publication ethics.

# 9. General breakdown of the costs and costs by phases of each year

| **S.no** | Phase | **Cost (birr)** |
| --- | --- | --- |
| 1. | Phase 1 | 1,149,379 |
| 2. | Phase 2 | 1,366,475 |
| 3. | Phase 3 | 120,230 |
| 4. | Grand total | 2,636,084 |

# References

Friso, F., Mendive, F., Soffiato, M., Bombardelli, V., Hesketh, A., Heinrich, M., ... & Politi, M. (2020). Implementation of Nagoya Protocol on access and benefit-sharing in Peru: Implications for researchers, Journal of ethnopharmacology, 259, 112885.

Sidik SM; Weaving Indigenous knowledge into the scientific method; Nature, 2022 Jan;601(7892):285-287. doi: 10.1038/d41586-022-00029-2. PMID: 35017707.

Kewessa, G., Abebe, T., & Demessie, A. (2015). Indigenous knowledge on the use and management of medicinal trees and shrubs in Dale District, Sidama Zone, Southern Ethiopia. Ethnobotany Research and Applications, 14, 171-182.

Asnake, S., Teklehaymanot, T., Hymete, A., Erko, B., & Giday, M. (2016). Survey of medicinal plants used to treat malaria by Sidama People of Boricha District, Sidama Zone, South Region of Ethiopia. Evidence-Based Complementary and Alternative Medicine, 2016.

Tefera, B. N., & Kim, Y. D. (2019). Ethnobotanical study of medicinal plants in the Hawassa Zuria District, Sidama zone, Southern Ethiopia. Journal of ethnobiology and ethnomedicine, 15(1), 1-21.CSA. (2019).

CSA: Population Projection of Ethiopia for All Regions At Wereda Level from 2014 – 2017. p 118, Federal Democratic Republic of Ethiopia Central Statistical Agency (CSA), Addis Ababa.

Pankhurst, R. (1990). *An Introduction to Medicinal History of Ethiopia.* Pp. 250-261. The Red Sea Press, Inc. New Jersey.

Pankhurst, R. (2001). The status and availability of oral and written knowledge on traditional health care. In: Zewdu, M., Demissie, A. (Eds.), Conservation and Sustainable Use of Medicinal Plants in Ethiopia Proceeding of The National Workshop on Biodiversity Conservation and Sustainable Use of Medicinal Plants in Ethiopia. IBCR, AA, pp. 92–106. 28 April-01 May 1998.

Kassaye KD, Amberbir A, Getachew B, Mussema Y. A historical overview of traditional medicine practices and policy in Ethiopia. Ethiopian Journal of Health Development. 2006;20(2):127-34.

Kewessa, G., Abebe, T., & Demessie, A. (2015). Indigenous knowledge on the use and management of medicinal trees and shrubs in Dale District, Sidama Zone, Southern Ethiopia. Ethnobotany Research and Applications, 14, 171-182.

# Theme One: Ethnobotany survey and Prevalence of ameessa plants use and Clinical Characterization of Feyancho Disease among Sidaama people

Menfes Tades, School of Natural Resources and Environmental Studies, Wondo Genet College of Forestry and Natural Resources, Hawassa University

Tsadiku Getachew, MD, Adaare General Hospital,

Maeregu Mulugeta, BSc in Chemical Engineering, Institute of Technology, Hawassa University,

Serawit Deyno, B. Pharm, M.SC, PhD, School of Pharmacy, Hawassa University

Sintayehu Fekadu, B. Sc, M.Sc, PhD, School of medical laboratory technology, Hawassa University, Hawassa, SNRS

Nigatu Tuasha, PhD in Biomedical Sciences, Hawassa College of Teachers Education

# Executive Summary

**Background**: Ethnobotany, study of the biological knowledge of particular ethnic groups, focuses on how plants have been or are used, managed and perceived in human societies and includes plants used for food, medicinal, rituals, social life and other purposes. Indigenous remains unexplored in many developing countries. Recently, ethnobotanical studies are gaining due attention globally and reasonably acceptable proportion of studies are being conducted in different parts of Ethiopia. In Sidaama, very few ethnobotanical studies were conducted and reported the use of traditional medicinal plants for the treatment of various health conditions. The present study aims to investigate the ethnobotanical knowledge of Sidaama people about the ameessa plants and the prevalence of health conditions of the infants treated by ameessa plants.

**Methods:** Ethnobotanical study will be conducted using reconnaissance survey and identification of informants from the sampling sites will be done. Floristic data will be collected, numbered and pressed carefully for identification of the plant species. Ethnobotanical data will be collected mainly by interview and observation-based field study. Careful recording of ecological data including altitude and geographical coordinates will be done using GPS at the spot during specimen collection. The prevalence of the illness of infants treated by the ameessa plants will be determined. Data entry validation, data quality assurance approaches and appropriate statistical techniques will be done.

The outcomes of the study will be published in reputable journals and due addressing of the results will be done to assure proper communication to the scientific community. Top rated ameessa plants will be scored and ethnopharmacological studies, efficacy testing bioassays and nutritional content profiling studies will be conducted in the next theme. In further theme, the safety study will be conducted using animal models. The latter two themes will be pursued subsequently and will be published upon completion. This phase of the study requires a total of 212,223.80birr for effective completion of the study.

*Key Words/Phrases*:	Ameessa, Ethnobotany, Indigenous knowledge, Sidaama, Traditional Medicine

# 1. Introduction

## 1.1. Background

Indigenous, or native, people have always had a significant relationship with the ecosystems in which they live. These ecosystems are vital for their survival, providing a wide array of plants for food, shelter, and tools. Those plants also provide sources for the medicines and religious rituals that are woven into the native cultures (Anderson, 2011). As the ecosystems provide a kind of revenue for indigenous people, they, in turn, serve as stewards of their environments and have done so for generations. The indigenous knowledge is, hence, the awareness and ability of native people to create practical and lifesaving products from their environments. It refers to the close interrelationship between the environment and aspects of their culture. Indigenous knowledge is an oral tradition that is shared within a native person’s own culture (Young, 2007).

It is important to acknowledge that many discoveries in the field of medicine were mainly based on traditional knowledge from a wide diversity of societies and cultures. Indeed, it is safe to say that currently accepted modern, allopathic medicine has its roots in traditional medicine and therapies (Mans, 2013). Notably, nearly 75% of the plant-derived prescription drugs used worldwide have been discovered following leads from local medicine (Wakdikar, 2004). Not surprisingly, the application of ancient wisdom and folk medicine remains an important strategy to discover and develop new drugs against human diseases.

It is the result of many generations’ long years’ experiences, careful observations and trial and error experiments (Martin, 1995). They are handed-over from one generation to the next verbally with great secrecy. Such secrete makes indigenous knowledge vulnerable to distortion and in most cases; some of the lore is lost at each point of transfer (Getahun, 1976). Therefore, there is a pressing need for systematic documentation of such useful knowledge through ethnobotanical research.

Feyancho diseases are well known among the people but it was not characterized by scientific methods by clinicians. The disease could possibly be infectious diseases. Infections caused by microorganisms have threatened human life since time immemorial. During the pre-antibiotic era, these have been a major concern for the high morbidity and mortality in humans. Some of the virulent organisms with the potential to spread infection from one infected person to another at a very rapid rate may cause worldwide pandemics, epidemics or outbreaks. With the discovery of the first antibiotic, "the magic bullet" Penicillin in the year 1943, patients could be effectively cured of many life-threatening infections. This gave a huge relief to the medical practitioners. Next three decades saw the development and discovery of a wide variety of antimicrobial agents. Subsequently, the pace of discovery of newer molecules declined from 1970 to 1987. It has reached a “discovery void” level from 1987 onwards up till now.

Feyancho disease has been a disease well known among Sidaama people for long years. The symptoms of the disease are well articulated in the community elders and they have been treating the disease by the local medicinal plants for the long period of time. As our knowledge is concerned there is no research done on the etiology of this disease. The only knowledge about the disease’s clinical manifestations is at the hand of the local communities because there is no documented information. Signs and symptoms, physical examination, clinical chemistry values, hematology, microbial profile, urine analysis, stool examination are not yet conducted. Therefore, this study will characterize the disease profile.

The present study derived its hypothesis based on the observation that ameessa plant is widely used among the Sidaama people to treat health conditions in infants. The collective name ameessa encompasses different plants used in various ways to treat the health condition. The present study, therefore, proposes that there are different ameessa plants traditionally used to treat health conditions in infants among sampled rural communities in Sidaama national regional state.

## 1.3 Conceptual Framework

**Initial Stage:**

Literature reading, proposal write-up, preparation for the fieldwork

**Reconnaissance survey: Contact and locate**

Elderly people, traditional healers, local administrators, respected/influential personalities and Development Agents/Farmer Training Center (DAs/FTC)

**Informant selection:** Using purposive sampling

Traditional medicine practitioners will be selected

**Ethnobotanical Data and Floristic Data Collection: Quality data collection**

-Interview and group discussion, guided field observation, voice and video recording, checklist, and Identification of Plant Species

Preferences ranking, Informants consensus factor fidelity levels

Qualitative and quantitative statistical analysis of ethnobotanical data

Data entry and validation

Priority plant species identification for further experimental consideration, Final report and Publication

Figure 1: Brief flowchart of the activities to be undertaken

# 2. Significance of the Study, Objectives, Statement of the Problem and Research Questions

## 2.1 Significance of the Study

Generally, the present study will have the following significances.

- Documenting valuable indigenous ethnobotanical knowledge of the rural communities about ameessa plant and salvage the decimation of the long-conserved traditional knowledge.
- Document the description of the health conditions of infants treated by ameessa plants and lay basis to relate western concepts of medicine.
- Recommending possible conservational approaches based on the status of endangerment of the ameessa plants in the study areas.
- Providing a platform for possible further investigations in the area.

Our study will also be the first study which is going to investigate the etiologic agent of feyancho disease. The findings of our research will have a profound importance in providing firsthand information regarding the etiology of feyancho disease. To the best level of our knowledge, there is no specific clinical manifestations that described the feyancho disease. Therefore, our finding will be the ground to effective diagnosis of the diseases condition, management of the diseases and optimization of the traditional management.

## 2.2 Objectives

### 2.2.1 General Objective

- To investigate and document the current status of traditional knowledge of ameessa plants and characterize health conditions of infants treated by it among Sidaama people

### 2.2.2 Specific Objectives

- To document ameessa plants and parts used by people of Sidaama people
- To assess the traditional formulations, dosages and duration of the ameessa treatment to infantile health condition
- To determine signs and symptoms of feyancho disease
- To determine possible microbial infection (viral, bacterial & parasitic) of the infantile health conditions
- To determine clinical chemistry profile of feyancho diseases
- To determine hematological condition related with diseases condition
- To perform urine analysis of patients with the disease’s conditions

## 2.3 Statement of the problem and Research Questions

Sidaama people are inhabitants in its present settlement for thousands of years now. In long historical establishment in the process, Sidaama people have their own concept of defining health and illness. The lives of Sidaama are highly interrelated with the nature and hence the people derive their basic needs for living and other necessities from the environment they live with. They also have special environmental protection cultures cultivated for long. Among immense valuable resources the Sidaama people have, traditional healthcare practices and herbal medicine remain untouched and practitioners are passing away with valuable indigenous knowledge, which could be indispensable with respect to health challenges of humanity these days. This includes their deep-rooted description of health conditions in infants and treatment modalities which is very precise and well established. There were, however, no well-coordinated research and documentation of such traditional medicinal practices among vast majority of rural residents of the SNRS. Therefore, the present study will answer the following main research questions.

- What are the medicinally useful ameessa plant species used to treat health conditions in infants by indigenous Sidaama peoples in the study areas?

- Which part of the plant is used for the treatment?

- What are the habitats and habit distribution of the plant species used as ameessa?

- What are methods of preparation, dosage, duration of treatment regimen and ways of administration, if other than drenching?

- What are sets of practices the local people employ to manage and conserve the ameessa plant species? What are the major threats ameessa plants around the study area?

- According to indigenous knowledge, which ameessa plant is the safest and efficacious one to treat the health conditions in infants?

- Which ameessa plant has high informant consensus factor and fidelity level?

# 3. Materials and Methods

## 3.1. Materials

The following materials will be used during the study.

- Digital camera and sound recorder
- Global Position System (GPS)
- Plant press, pressing materials and other herbarium equipment
- Plastic bags
- Field bags, raincoat and field shoes
- Field notebook, pens and pencils, CDs, Flash discs
- Laptop computer and necessary software

## 3.2. Study design

This is a field survey and a case study of a selected case.

## 3.3. Sample collection Area: the Community

The proposed ethnobotanical study will be conducted in nine (9) Woredas (Districts) of the SNRS stratified based on the ecological climatic zones of the region. The SNRS is the 10^th^ member state of the Ethiopian Federal Democratic Republic officially formed after the Referendum conducted on November 20, 2019 (Figure 1). Formerly one of the highly populous zones under the Southern Nations, Nationalities and Peoples regional state, Sidaama accounts for 4.0% of the National population of about 115 million (CSA, 2019). Administratively, the region is structured into 30 woredas (districts) and seven city administrations. The capital city of the SNRS is Hawassa city and is 275km away from Addis Ababa in the southern direction with the projected population of half a million (CSA, 2019).

Sidaama has a variety of climatic conditions. Warm conditions cover 54% of the area. Locally known as Gammoojje or Woinadega, is a temperate zone ranging from an elevation of 1500 m to 2500 m above sea level with the mean annual rainfall between 1200 mm and 1599 mm & 15°C to 19.9°C average annual temperature. A hot climatic zone, Kolla, covers 30% of the total area and has an elevation range from 500 m to 1500 m above sea level with a mean annual rainfall of 400 mm to 799 mm, and the mean annual temperature ranges from 20°C to 24.9°C. Cool climatic conditions known as Alichcho or Dega exists in the mountainous highlands. This covers 16% of the total area with an elevation between 2500 m and 3500 m above sea level. This part gets the highest amount of rainfall, ranging from 1600 mm to 1999 mm. It has a mean annual temperature of 15°C to 19.9°C. Thus, the nine woredas (districts) where the present study will take place are: Borricha, Daraara and Doore woredas representing a hot climatic zone; Haweela, Daalle, Daarra woredas from a warm climatic zone and Hula, Bursa and Harbagoona from cold climatic zone. Two kebeles from each woreda will be sampled by a lottery method.

Figure 2. The map showing the geographical location of the SNRS and the study sites (Source: Google map).

## 3.4. Reconnaissance survey

A reconnaissance survey in the study area will be conducted ahead of the actually ethnobotanical survey. During the reconnaissance survey, administrators of the woreda/kebeles and elderly people resided in the area for longer time will be consulted on how to locate and approach traditional medicine practitioners found in the locality, with especial reference to ameessa knowledge. As there could be reluctance to reveal the ameessa plant used for the treatment, sons/daughters/relatives of the traditional practitioners, when applicable, will be approached and negotiations may be employed to achieve the end point. Farmer Training Centers (FTCs), health/agricultural extension workers of the area will be consulted during the reconnaissance survey to identify and approach the traditional practitioners the ameessa knowledge being a priority. During this period, the characteristic description of the health conditions in infants treated with the ameessa plant will be investigated.

## 3.5. Informant selection

Since this is a purposive investigation, the traditional practitioners with the ethnobotanical knowledge about ameessa plant will be identified during the reconnaissance survey. Therefore, the local residents of the study area with the knowledge of ameessa plant and offer traditionally prepared treatment to health conditions in infants will be major informants to this study. To record knowledge held by traditional healers, the choice of informants will be dictated (Martin, 1995).

## 3.6. Ethnobotanical Data Collection

Ethnobotanical data collection requires a systematic approach and information can be collected through actual field observation and semi structured interviews depending on the particular objective of the research taking into account Bennett’s Golden Rules (McClatchey and Gollin, 2005) for ethnobotany fieldwork. Therefore, interview and observation-based field study will constitute the main data collection methods for this particular study. Standard format (appendix 1 and 2) based on methods given by Hedberg (1993), Martin (1995) and Cotton (1996) will be used to record the information while the traditional healers’ detailed explanation about the ameessa plant(s) used, description of the infant’s health condition treated and how the remedy is prepared and delivered will be documented. In addition, voice records and video recorder will be employed to cross check the recording on the prepared formats. The checklist will be translated to Sidaamu-Afoo (appendix 3) before interviewing and discussing with traditional medicine practitioners. Interviews and discussions will be held in Sidaamu-Afoo directly by the investigators. Ethnobotanical data from and around home gardens will be collected following Vogl *et al.* (2004) to retrieve the indigenous botanical knowledge of male and female informants.

## 3.7. Floristic Data Collection and Identification of Plant Species

Careful recording of ecological data including altitude and geographical coordinates will be done using GPS at the spot during specimen collection. The collected plant specimens will be numbered, pressed and dried at the field properly for identification. Appropriate documentation will be done for necessary photographic pictures taken along with specimens during data collection. The plant material will be taken to the National Herbarium, Department of Plant Biology and Management, College of Natural Science, Addis Ababa University (AAU) for identification, authentication and final deposition.

## 3.8. Determination of the prevalence of infantile illness

During the field data collection of ameessa plants (both ethnobotanical and floristic), household sample units will be studied to determine the prevalence of infantile health conditions treated by ameessa plants. The prevalence determination will be based on the number of total births in the sampled households. Briefly, the presence of attribute in the general population is estimated to be at 50%. Thus, the sample size will be calculated based on single population proportion formula by taking the margin of error of 0.05 at 95% confidence level, and then the calculated sample size will be 384. Adding a 10% non-response rate, the total household unit to be sampled will be 422.4.

n= (Zα/2)^2^p (1-p)/d^2^	= (1.96)2 * 0.5(1-0.5)/ (0.05)2

=3.84*0.25/0.0025=384

Zα/2 = 1.96, (Zα/2)^2^=3.84

P = 50% (no previous study)

d = 0.05, (0.05)^2^= 0.0025 (desired precision)

10% non-response rate = 38.4

N =384+38.4 = 422.4

The prevalence of the infantile health conditions in the SNRS will be determined during theme while surveying the ethnobotanical data. During this investigation, details of peoples’ perceptions and definitions of the feyancho disease will also be documented. Part of the data generated during this time will be augmented with the clinical characterization of the feyancho disease.

## 3.9. Eligible criteria of the participants in a case study

- Patients with age of <5 years who has disease manifestations will be included in the study.
- Only client whose caregiver has given written informed consent and has a disease during the study period will be included in the study.
- Participants out of study period and those whose caregivers won’t be volunteered will not be included in this study.
- Clients who are critically ill and are of caregivers who had psychiatric problem also will not be selected. .

## 3.10. Data collection

A pretested and structured questionnaire will be used to collect socio-demographic, associated factors data. Blood, stool, urine and specimens from affected area will be taken from each of patient with sterile material using the standard procedure. Physical examination will be conducted by physicians. The questionnaire will be filled by trained clinical nurses. Specimens will be collected at the same day of questionnaire data collection by laboratory technologists for each under five patient at three kebeles of Wondogenet, Melga and Yirgalem Woredas.

Swabs, stools, urines and blood will be collected from the participants. Samples will be collected from each study participant and processed by laboratory technologist. The sample will be processed and analyzed in Hawassa University comprehensive specialized hospital and SNNPR/PHIL. Urine analysis, clinical chemistry, hematology, stool examination will be conducted using the available standards and instruments. Standard operating procedure will be followed for each step. Bacterial culturing will be done and pathogens will be confirmed by using characteristics growth on primary culture media, morphology and identification of gram positive and gram negative, and biochemical test.

## 3.11. Data Analysis

Data entry and validation will be done using Graphpad prism version 5 and every possible data quality assurance approaches will be following throughout the study period. Finally, the validated data will be analyzed through the application of both qualitative and quantitative ethnobotanical methods as well as all possible statistical techniques. Accordingly, descriptive statistics, preference ranking, paired comparison, informant consensus and fidelity level tests will be done as follows.

**A. Descriptive statistics**

Descriptive statistical methods such as percentages and frequency will be employed to analyze and summarize the data on ameessa plants, use and associated knowledge. In order to determine the proportions of descriptive statistic, simple calculation, bar graphs will be employed.

**B. Preference ranking**

This will be done according to Martin`s procedure (1995). Selected ameessa plants believed to be most effective to treat health condition in infants will be given the highest value (5) and the least effective will be given the lowest value (1). The value of each species will be summed up and the rank for each species will be determined based on the total score. This will be done to indicate the most effective ameessa plant used by the community to treat the health condition in infants.

**C. Paired comparison**

Paired comparisons will be used to evaluate the degree of preference or levels of importance of certain selected medicinal plants. To find out about the effectiveness and popularity of five ameessa plant species used to treat health condition in infants which are frequently used in the study area, randomly paired comparison will be done according to Martin, (995). To do this, five (5) key informants from each site will be allowed to compare the randomly paired sequenced ameessa plants. Then, their responses on the value of each medicinal plant will be recorded and in accordance with their responses, the total score of the key informants on each medicinal plant will be summed and ranked accordingly. Finally, the highest score of a plant, the highest rank and the more effective medicinal plants will be described.

**D. Informant consensus**

In order to evaluate the reliability (consistency of the information on a particular subject) and validity (accuracy of the information), each informant will be contacted and interviewed at least two times for the same ailment and traditional medicines used. Checking for implausibility of statements and crosschecking data with and between informants will be done according to Alexiades (1996). If the idea of an informant contradicts during the second or later discussion on the same issue, it will be rejected. To identify the agreements of the informants on for identifying potentially effective ameessa plant used for the treatment of health condition in infants, informant consensus factor (F_ic_) will be calculated for each category. Thus, F_ic_ will be calculated as follows: number of use citation in each category (n_ur_) minus the number of species used (n_t_), divided by the number of use citations in each category minus one (Trotter and Logan, 1986 as in Heinerich, 2000).

Where: F_ic_: Informant Consensus Factor

n_ur_: number of use-reports

n_t_: number of taxa (esp. species) used

The outcome ranges from 0 to 1. A high value (close to 1) indicates that relatively few taxa (usually species) are used by a large proportion of the healers, while a low value indicates that the informants disagree on the taxa to be used in the treatment within a category of illness (Heinerich*,* 2000).

**E. Fidelity level**

The percentage of informants claiming the use of a certain plant for the same major purpose, fidelity level (FL), will be calculated for the most frequently reported diseases/ailments as:

FL (%) = $\frac{Np}{N}\times100$

Where, Np is the number of informants that claim a use of a plant species to treat a particular disease, and N is the number of informants that use the plants as a medicine to treat any given disease (Alexiades, 1996).

# 4. Description of the action and its effectiveness

The main actions to be undertaken under this theme are mobilizing resources to collect ethnobotanical and floristic data, document the priceless traditional ameessa medicinal plants knowledge of the community before it is decimated, document the definitions/characteristics of the health conditions of infants treated by ameessa and identify priority ameessa MPs for further in-depth bioassays using ethnobotanical tools.

We will document the definitions/characteristics of the health conditions of infants treated by ameessa as described by the practitioners in the study area. In addition, clinical samples will be collected and analyzed to determine the etiologic agent of the disease in infantile. Here, both disease definitions of the practitioners collected during the ethnobotanical survey and clinical evaluation in the lab will help depict the etiology and guide further bioassay following identifying the etiology. The completed study will be published in a reputable international journal. The finding will be disseminated to the audience through proper means.

# 5. Expected output

A majority of the traditional knowledge maintained by transferring from one generation to the other through oral dictation is at the verge of extinction as it is covered up by strict secrecy. Most of the time, the age of elderly parents come to an end before transferring the knowledge the entrusted older son. Though some TMPs knowledge seems to be in public domain, it should not be taken for granted that it vanishes in time as population ages. Therefore, documenting the TMPs knowledge properly is the only reliable way to salvage the knowledge. The endpoint of this ethnobotanical survey (theme one) will be publishing the finding in the reputable international journal and prioritize ameessa plants for an in-depth bioassays.

The disease/health condition in infants treated by the diseases will be defined based on empirical evidence generated through scientific investigation. This will lay foundation to plan and undertake proper experimental assays to test the efficacy of the ameessa bioactive principles/compounds/extracts. The findings will be published in the prestigious reputable international journal.

# 6. Beneficiaries

Nowadays, ameessa plant is not only used by a rural community. It is widely practiced in urban settings. Therefore, a proper documentation of the medicinal plants used as ameessa benefits rural and urban community of the SNRS.

# 7. Ethical Clearance

Ethical clearance will be obtained from the Ethical Review Committee of Hawassa University. Permission will be obtained from the Sidaama regional state, woreda administrators and kebele officers. Written or informed consent will be obtained from study participants after a brief explanation about the objectives of the study to the participants. Confidentiality will be kept carefully.

# 8. Monitoring and Evaluation

Internally, the researchers will carry out the project with strict research ethics and will disclose relevant information about the conduct to the concerned quality assuring body upon reasonable request. The team will evaluate the progress of the project periodically and will document the minute of the meeting. External authority, delegates of the research and technology transfer office, may convene a relevant monitoring and evaluation meetings. The progress report and final report of the project, upon completion, will be duly communicated as per scheduled by the office. Finally, the publication will be produced following proper publication ethics.

# 9. Work Plan

| **S. No** | **Main activity** | **Time/months the activities will be accomplished** | | | | | | | | | | | | | | | | | | | |
| --- | --- | --- | --- | --- | --- | --- | --- | --- | --- | --- | --- | --- | --- | --- | --- | --- | --- | --- | --- | --- | --- |
|  |  | **2021-2022** | | | | | | | | | | | | | | | **2023** | | | | |
|  |  | Dec. | Jan. | | Feb. | Mar. | April | | May | June | July | | Aug. | Sep. | Oct. | Nov. | Jan | Feb | Mar | Apr | May |
| 1 | Title selection, | ✓ | ✓ | |  |  |  | |  |  |  | |  |  |  |  |  |  |  |  |  |
| 2 | Proposal development | ✓ | ✓ | |  |  |  | |  |  |  | |  |  |  |  |  |  |  |  |  |
| 3 | Reconnaissance survey |  |  | | ✓ | ✓ |  | |  |  |  | |  |  |  |  |  |  |  |  |  |
| 4 | Informants selection |  |  | |  | ✓ | ✓ | |  |  |  | |  |  |  |  |  |  |  |  |  |
| 5 | Ethnobotanical Data collection |  |  | |  |  | ✓ | | ✓ | ✓ |  | |  |  |  |  |  |  |  |  |  |
| 6 | Floristic Data Collection |  |  | |  |  |  | | ✓ | ✓ | ✓ | | ✓ |  |  |  |  |  |  |  |  |
| 7 | Identification & authentication |  |  | |  |  |  | |  |  |  | | ✓ | ✓ |  |  |  |  |  |  |  |
| 8 | Data analysis &write up |  |  | |  |  |  | |  |  |  | | ✓ | ✓ | ✓ |  |  |  |  |  |  |
| 9 | Final report |  |  | |  |  |  | |  |  |  | |  |  | ✓ |  |  |  |  |  |  |
| 10 | Public dissemination |  |  | |  |  |  | |  |  |  | |  |  |  | ✓ |  |  |  |  |  |
| 11 | Manuscript preparation |  |  | |  |  |  | |  |  |  | |  |  |  | ✓ |  |  |  |  |  |
| Clinical characterization study | | | | | | | | | | | | | | | | | | | | | |
| 12 | Ethical review |  | |  |  | |  |  | | | |  |  | | | | ✓ |  |  |  |  |
| 13 | Field survey |  | |  |  | |  |  | | | |  |  | | | |  | ✓ |  |  |  |
| 14 | laboratory analysis |  | |  |  | |  |  | | | |  |  | | | |  | ✓ | ✓ |  |  |
| 15 | Data analysis & report write up |  | |  |  | |  |  | | | |  |  | | | |  |  | ✓ |  |  |
| 16 | Revision and finalizing the report |  | |  |  | |  |  | | | |  |  | | | |  |  | ✓ | ✓ |  |
| 17 | Preparing for public dissemination |  | |  |  | |  |  | | | |  |  | | | |  |  |  | ✓ |  |
| 18 | Manuscript preparation |  | |  |  | |  |  | | | |  |  | | | |  |  |  |  | ✓ |

# 10. Cost of the Study and Budget Breakdown

Table 1: **Budget Breakdown**

| **S. №** | **Description of major expenses** | **Meas. Unit** | **Quantity** | | **Unit price (EtB)** | **Total price (EtB)** | **Remark** |
| --- | --- | --- | --- | --- | --- | --- | --- |
| **Part 1** | **Materials and Equipment** |  |  |  |  |  |  |
| 1.1 | GPS (GARMlN GPS 72HGPS) | **№** | 1 | | 17,750.00 | 17,750.00 | If the university provides the GPS, this cost can be exempted |
| 1.2 | Canon EOS Rebel T2i Digital SLR Kit w/ EF-S/18-55 IS II | **№** | 1 | |  |  | Personal camera will be used |
| 1.3 | Plant Press | **№** | 2 | | 1,500.00 | 3,000.00 |  |
| 1.4 | Laptop computer (at least Core i5) | **№** | 1 | | - | - | Personal lap top will be used |
| 1.5 | Flash disc (SANDISK 128GB iXpand Flash Drive Go USB 3.0 Lightning iPhone SDIX60N-128G-GN6NE) | **№** | 1 | | 3,577.00 | 3,577.00 |  |
| 1.6 | Plastic Bags | **№** | 50 | | 15.00 | 750.00 |  |
| 1.7 | Raincoat | **№** | 3 | | 2,500.00 | 7,500.00 |  |
| 1.8 | Pair of Field shoes | **№** | 1 | | 1,500.00 | 1,500.00 |  |
| 1.9 | Field bags | **№** | 3 | | 1,500.00 | 4,500.00 |  |
| **Subtotal of Part 1** | | | | | | **38,577.00** |  |
| **Part 2** | **Transport cost and per diem** | **Trip/**  Round/rate | **Quantity** | | **Unit price (EtB)** | **Total price (EtB)** | **Remarks** |
| 2.1 | Fuel | 18 | 40 | | 28.94 | 20,836.80 |  |
| 2.2 | Lubricant | 3 | 1 | | 2500 | 7,500.00 |  |
| 2.3 | Professional payment for tool development | 10 | 3 | | 500.00 | 15,000.00 |  |
| 2.4 | Per diem during reconnaissance survey | 20 | 3 | | 339.00 | 20,340.00 |  |
| 2.5 | Per diem for ethnobotanical data collection | 30 | 3 | | 339.00 | 30,510.00 |  |
| 2.6 | Per diem for floristic data collection | 10 | 3 | | 339.00 | 10,170.00 |  |
| 2.7 | Per diem for the driver | 30 | 1 | | 249.00 | 7470.00 |  |
| 2.8 | Field guide | 10 | 9 | | 249.00 | 22,410.00 |  |
| 2.9 | Key informants | 5 | 18 | | 249.00 | 22,410.00 |  |
|  | Professional payment of taxonomists for the species identification and authentication |  |  | |  | 15,000.00 | Depends on the number of the spp for identification |
|  | Perdium for herbarium registration | 3 | 20 | | 650 | 39000 |  |
| Part 4 | Stationery packages (notepads, pen, pencil, photocopying, prints and …) | | | Gross amount allocated | | **2,000.00** | May be exempted |
| **Budget for clinical characterization** | | | | | | | |
| Payments for Personnel | | | | | | | |
|  | Laboratory technician | | | 8 person | | **450*22** | 79200.00 |
|  | Sample collectors | | | 4 person | | 450*22 | 39,600 |
|  | Supervisors | | | 4 person | | 450*22 | 39,600 |
|  | Health extension workers | | | 6 person | | 450*22 | 59,400 |
|  | Cleaner | | | 2 person | | 450*22 | 19,800 |
| Laboratory Material and Reagents | | | | | | | |
|  | Test tubes(glass) | | | 500 pcs | | 5 | 2500 |
|  | Vacuum blood collection tube (5-10ml) | | | 5 box | | 300 | 1500 |
|  | Microscopic glass slides | | | 5 pk | | 500 | 2500 |
|  | Urine collection tube | | | 4 pk | | 200 | 800 |
|  | Stool collection cap | | | 4 pk | | 200 | 800 |
|  | Physiological saline solution | | | 2 bottle | | 200 | 400 |
|  | Gram stain solution | | | 1 bottle | | 2000 | 2000 |
|  | Formaldehyde (for stool concentration) | | | 1 bottle | | 1000 | 1000 |
|  | Ether (for stool concentration) | | | 1 Bottle | | 1000 | 1000 |
|  | Bacterial culture basic media | | | 5 Bottle | | 3500 | 17500 |
|  | Bacterial culture differential media | | | 20 Bottle | | 3500 | 70000 |
|  | Ice box (for sample transportation) | | | 2 pcs | | 2000 | 4000 |
|  | Rotavirus detection ELISA kit | | | 4pk (of 25 each) | | 30,000 | 120,000 |
|  | Swab of 100 | | | 4 bag | | 150 | 600 |
|  | Catalase | | | 1 Litter | | 200 | 200 |
|  | Coagulase | | | 20 Vial | | 220 | 4400 |
|  | Biohazard bag | | | 4 pcs | | 50 | 200 |
|  | Glove of 20 | | | 4 pcs | | 100 | 4000 |
|  | Towel paper of 1000 | | | 2 Carton | | 50 | 250 |
|  | Crimper | | | 02 pcs | | 14000 | 28000 |
|  | Aluminum (20mm) of 100 | | | 3 pk | | 10500 | 31,500 |
|  | Flash disk | | | 1 pcs | | 200 | 200 |
|  | Slide of 50 | | | 8 pk | | 80 | 640 |
|  | Domestic bleach | | | 2 bottle | | 50 | 100 |
|  | Permanent marker | | | 5 pcs | | 20 | 100 |
|  | Registrations book | | | 1 pcs | | 50 | 50 |
|  | Cryovials, sterile, external threads, 2ml | | | 200 pcs | | 1 | 200 |
|  | Ethanol (absolute) | | | 1litre | | 200 | 200 |
|  | 70% alcohol | | | 1 liter | | 150 | 150 |
|  | Cotton | | | 3 roll | | 200 | 600 |
|  | Petri dish | | | 10pcs | | 200 | 2000 |
|  | Transportation cost | | | Lump sum | | | 50000 |
|  | **Contingency** | | |  | |  | **100943** |
|  | **Total** | | |  | |  | **1,149,379** |

# References

Alexiades, M.N. (1996). Collecting Ethnobotanical Data: An Introduction to Basic Concepts and Techniques Selected Guidelines for Ethnobotanical Research: A Field Manual, 53-94. Scientific Publications Department, New York, Botanical Garden ,Bronx, New York.

Anderson, E.N. (2011). Overview of a Growing Field Ethnobiology*. In Ethnobiology*. (Anderson E. N., Pearsall, D., Hunn E., and Turner N., eds) Wiley-Blackwell. Published John Wiley and Sons, Inc

Awas, T., Gashaw, M., Tesfaye, G. and Tihune, A. (2003). *Ecosystems of Ethiopia.* National Biodiversity Strategy and Action Plan (NBSAP) Project, IBCR, Addis Ababa, Ethiopia

Bekele, E. (2007). Study on Actual Situation of Medicinal Plants in Ethiopia. *Prepared for*: Japan Association for International Collaboration of Agriculture and Forestry

Belayneh A, Asfaw Z, Demissew S, Bussa N (2012). Medicinal plants potential and use by pastoral and agropastoral communities in Erer Valley of BabileWereda, Eastern Ethiopia. Journal of Ethnobiology and Ethnomedicine 8:42.

Cohen ML. Changing patterns of infectious disease. Nature **2000**; 406:
762–7.

Cotton, C.M. (1996). *Ethnobotany.Principles and Applications*. John Willey and Sons, Chichester, UK.

CSA. (2019). Population Projection of Ethiopia for All Regions At Wereda Level from 2014 – 2017. p 118, Federal Democratic Republic of Ethiopia Central Statistical Agency (CSA), Addis Ababa.

Dawit A (2001). The role of medicinal plants in healthcare coverage of Ethiopia, the Possible Integration.In:Conservation and Sustainable Use of Medicinal Plants in Ethiopia. (MedhinZewdu and DawitAbebe (eds). Proceeding of The National Workshop on Biodiversity Conservation and Sustainable Use of Medicinal Plants in Ethiopia, 28 April-01 May 1998, IBCR, Addis Ababa, Ethiopia. pp 6-21

Debela, A., Abebe, D. and Urga, K. (1999). An over view of traditional medicine in Ethiopia: perspective and developmental efforts. *In:* *Ethiopian Pharmaceutical Association.* Silver Jubilee Anniversary, Pp. 25-61. (Tamrat Ejigu, ed.). Addis Ababa, Ethiopia.

Desta, B. (1984): Ethiopian Traditional Herbal Drugs. Potentiality and Appropriate Utilization. Proceedings of the 8^th^ International Conference of Ethiopian Studies, pp.763 –766.

Ensermu K, Sebsebe D, Zerihun W, Edwards (1992). Some threatened endemic plants of Ethiopia. In: The Status of Some Plants in Parts of Tropical Africa.(Edwards, s. and ZemedeAsfaw (eds). Botany 200, NAPREC, Monograph series number 2:35-55. Addis Ababa University, Ethiopia.

Garrett L. The coming plague. New York: Farrar, Straus & Giroux,**1994**

Getahun, A. (1976). Some Common Medicinal and Poisonous Plants Used in Ethiopia Folk Medicine. Addis Ababa University, Addis Ababa. pp. 3-63.

Hedberg, I. (1993). Botanical methods in ethnopharmacology and the need for conservation of medicinal plants. *J. Ethnopharmacol*. 38:121-128.

<http://www.etflora.net> - accessed on May 2, 2014.

Jansen, P.C.M. (1981). Spices, Condiments and Medicinal plants in Ethiopia: Their Taxonomic and agricultural significance. Centre for agricultural publishing and documentation. Wageningen, The Netherlands.

Joint United Nations Programme on HIV/AIDS (UNAIDS). AIDS epidemic update: December 2000. Geneva: World Health Organization, **2000**

Kass EH. History of the specialty of infectious diseases in the United
States. Ann Intern Med **1987**; 106:745–56

Krause RM. The restless tide: the persistent challenge of the microbial
world. Washington, DC: National Foundation for Infectious Diseases,**1981**

Lederberg J. Infectious disease as an evolutionary paradigm; Emerg
Infect Dis **1997**; 3:417–23.

Mans, D.R.A. (2013). From forest to pharmacy: Plant-based traditional medicines as sources for novel therapeutic compounds. *Acad. J. Med. Plants*. 1(6):101-110.

Martin, G.J. (1995). *Ethnobotany: A method Manual*. Chapman and Hall, London. Pp. 265-270.

McClatchey, W. and Gollin, L.X. (2005). An Ethnobotany Research Training Workshop in Madagascar. Ethnobot. Res. Appl. 3:309-327. www.ethnobotanyjournal.org/vol3/i1547-3465-03-309.pdf.

Mesfin F, Seta T, Assefa A (2014). An ethnobotanical study of medicinal plants in AmaroWoreda, Ethiopia.Ethnobotany Research and Applications 12:341-354.

Misrak Bekele Areda**,** Zemede Asfaw and Gemedo Dalle (2014). Ethnobotanical Study of Medicinal Plants in Gorichei Wereda, Sidaama Zone, SNNPR, Ethiopia. Unpublished MSc Thesis, Addis Ababa University.

Mutke, J., Kier, G., Braun, G., Schultz, C. and Barthlott, W. (2001). Patterns of African vascular plant diversity – a GIS based analysis. *Syst. Geog. Pla.* 71:1125-1136.

Nguta JM, Mbaria JM, Gakuya DW, Gathumbi PK, KiamaSG (2010).Antimalarial herbal remedies of Msambweni, Kenya. Journal of Ethnopharmacolology 128(2):424-432.

Pinner RW, Teutsch SM, Simonsen L, et al. Trends in infectious diseases
mortality in the United States. JAMA **1996**; 275:189–93.

Rates, S.M.K. (2001). Plants as source of drugs. *Toxicon*. 39: 603–613.

Regassa, R. (2013). Diversity and conservation status of some economically valued indigenous medicinal plants in Hawassa College of Teacher Education Campus, Southern Ethiopia. *IJAR, 1(*3):308-328.

Sintayehu Tamene Beyene, Zemede Asfaw and Sebsibe Demissew (2011). An Ethnobotanical Study of Medicinal Plants in Wondo Genet Natural Forest and Adjacent Kebeles, Sidama Zone, SNNP Region, Ethiopia. Unpublished MSc Thesis, Addis Ababa University.

Tena Regassa, Zemede Asfaw and Ensermu Kelbessa, (2014). Endemic Medicinal Plants of Ethiopia: Review of the Literature. Addis Ababa University,Unpublished PhD Seminar paper

The world health report 2000—health systems: improving performance. Geneva: World Health Organization, **2000**

Tuasha, N., Petros, B. and Asfaw, Z (2018a). Medicinal plants used by traditional healers to treat malignancies and other human ailments in Dalle District, Sidama Zone, Ethiopia. *J. Ethnobiol. Ethnomed.* 14(1):15. https://doi.org/10.1186/s13002-018-0213-z.

UNEP (1995). Global biodiversity assessment, United Nations Environment Programme

Vogl, C.R., Lukasser, B.V. and Puri, R.K. (2004). Tools and Methods for data collection in Ethnobotanical Studies of Homegardens. *Field Methods* 16 (3): 285-306.

Wakdikar, S. (2004). Global health care challenge: Indian experiences and new prescriptions. *Electr. J. Biotechnol*. 7:217-223.

Young, K.J. (2007). Ethnobotany. *In: Green World Series* (Hopkins, W.G. ed) Infobase Publishing. Chelsea House publishers

# THEME TWO: BIOACTIVITIES OF SELECTED AMEESSA PLANTS AND DETERMINATION OF NUTRITIONAL VALUES

BY

Sintayehu Fekadu, B. Sc, M.Sc, PhD, School of medical laboratory technology, Hawassa University, Hawassa, SNRS

Menfes Tadesse, School of Natural Resources and Environmental Studies, Wondo Genet College of Forestry and Natural Resources, Hawassa University

Mairegu Daniel, BSc in Chemical Engineering, IoT, Hawassa University, Hawassa

Tsadiku Getachew, MD, Adaare General Hospital, Hawassa

Nigatu Tuasha, PhD in Biomedical Sciences, Hawassa CTE, Hawassa

Serawit Deyno, PhD, School of Pharmacy, Hawassa University

# 1. Introduction

## 1.1. Background

Medicinal properties of plants have been recognized since time immemorial. They are still used in medicine, food and cosmetic industry (Lahlou, 2004). Knowledge on plant uses is the result of many years of man’s interaction and selection on the most desirable, the most vigorous and the most successful plant present in the immediate environment at a given time (Roger, Pierre-Marie, Igor, & Patrick, 2015). According to the World Health Organization, 80% of people in developing countries still depend on local medicinal plants to fulfill their primary health needs (Burton, Smith, & Falkenberg, 2015).

The diseases that have been managed successfully using herbal medicine include malaria,
epilepsy, infertility, convulsion, diarrhea, dysentery, gonorrhea, flatulence, tonsillitis,
bacterial and fungal infections, mental illness and worm infections (Sofowora, 1996). There is a global consensus on the benefits of phytopharmacy and at present medicinal plants occupy a key position in plant research and medicine. In many African countries, such as Ghana, Mali, Nigeria and Zambia, the first line of treatment for 60% of the children with high fevers, is the use of herbal medicines at home (World Health Organization International Society of Hypertension Writing Group, 2003). The importance of plants in medicine remains even of greater relevance with the current global shift to obtain drugs from plants sources, as a result of which attention has been given to the medicinal value of herbal remedies for safety, efficacy and economy (Aliyu, Musa, Abdullahi, Ibrahim, & Oyewale, 2011).

In Ethiopia, traditional remedies represent not only part of the struggle of the people to fulfill their essential drug needs but also they are integral components of the cultural beliefs and attitudes (. In Ethiopia, about 800 species of plants, which occur throughout the country's diverse highlands and lowland areas, are used in the traditional health care system to treat nearly 300 physical and mental disorders and remains to be the main resource of treatment for a large majority of other diseases conditions (Giday, Asfaw, Elmqvist, & Woldu, 2003; Giday et al., 2007).

## 1.2. Objective of the study

- To evaluate pharmacological activities of selected Ameessa plants

### 1.2.1. Specific objectives

- To determine antibacterial activities of selected Ameessa medicinal plants
- To determine antifungal activities of selected Ameessa medicinal plants
- To isolate most active antibacterial agents from Ameessa plants
- To isolate most active antifungal agents from Ameessa plants
- To analyze the protein, fat, vitamin, zinc, iron, iodine, B-vitamins content of *Amessa* leaf extract

## 1.3. Significance of the study

According to the World Health Organization (World Health Organization International Society of Hypertension Writing Group, 2003), medicinal plants would be the best source to obtain a variety of drugs. Thus, scientific research on the healing properties and bioactivity of natural products especially of plant origin has been extensive particularly in the western world. However, a rich heritage of floral biodiversity is found in developing countries. For instance, Ethiopia, a country with a strong history of traditional healing hosts around 7,000 species of higher plants, of which 10-12% are endemic to the country. A large proportion of these plants are used for the treatment of infectious diseases including those caused by bacteria and fungi, which account for approximately one-half of the deaths in tropical countries (Lulekal et al., 2014). The aim of the present study is to determine ethnopharmacological properties of Ameessa medicinal plants used for the management of diseases in Sidama region.

# 2. Methods and materials

## 2.1. Plant extraction

Parts of the collected plants will be separated, washed carefully with tap water, rinsed with distilled water; shade dried and will be ground in to powder and stored at room temperature. The dried powder of the plants part will be taken in separate conical flasks and then solvent (80% methanol (MeOH) in H2O) will be added to the Erlenmeyer flask. The flasks will be kept at room temperature, shaking on a rotary water bath shaker (DZK-2, Shanghai, China) (120 routes per minute) for 72 h at ambient temperature with intermittent gently shaking. The extracts will be then filtered by using Whatman filter paper № 1 and Removal of the solvent and concentration will be done using a rotary vacuum evaporator (BÜCHI-Germany) under reduced pressure at 45°C. The concentrated extract will then be freeze-dried by lyophilization (CHRIST, Alpha 2-4 LDplus, *Osterode, Germany*)(Sasidharan, Chen, Saravanan, Sundram, & Latha, 2011).

## 2.2. Preliminary Phytochemical Screening Tests

Ferric chloride test: The extract will be prepared and then few drops of 5% ferric chloride solution will be added. A dark green color indicates presence of phenolic compounds.

Test for terpenoids: About 2ml of the extract solution is mixed with 2 ml of chloroform and 2ml of concentrated sulfuric acid. Formation of grey color shows terpenoid entity.

Test for alkaloids (Dragendoriff’s test): 1ml of 1% hydrochloric acid will be added to 2 ml of the extract solution followed by few drops of dragendoriff’s reagent which produces an orange or orange-red precipitate.

Test for flavonoids (Shinoda test): Pieces of magnesium ribbon and concentrated HCl will be mixed with plant extract solution and after few minutes pink color shows presence of flavonoid.

Test for glycosides (Keller-Killiani test): Test solution will be treated with few drops of glacial acetic acid and ferric chloride solution and mixed. Concentrated sulfuric acid will be added, and observed for the formation of two layers. Lower reddish brown layer and upper acetic acid layer which turn bluish green indicate a positive test.

## 2.3. Isolation by Chromatographic Techniques

Gradient solvent systems (non-polar to highly polar solvent) will be prepared and tested for best separation of various organic constituents. A column packed with silica gel 60 (Merck, high-purity grade, pore size 60 Å, 230 - 400mesh ASTM) will be conditioned with hexane slowly from the top. When a column is ready, the sample will be loaded inside the top of the column. The column will be separated from the incoming mobile phase by a thin layer of cotton to prevent the disturbance of the column. The mobile solvent will then be allowed to flow down through the column.

Weighed dried residue will be adsorbed to silica gel and separated on a cylindrical glass column (300 mm height × 22.5 mm diameter). The elution will be performed starting from hexane: ethyl acetate (10:0, 10:1, 5:1, 1:1, 1:5, 1:10) followed by mixture of ethyl acetate: methanol (10:0, 10:1, 5:1, 1:1, 1:5, 1:10, 0:10) of 100 ml each. Fractions will be collected and analyzed using TLC. Compound bands/spots visualized on TLC by visual detection, under UV light (254 nm and 360), in iodine chamber, and using spray reagent (30% sulfuric acid) for the presence of specific compounds. The visualized spots will be marked and the Rf value of each spot will be calculated. The Rf value is equal to the distance traveled by the compound divided by the distance traveled by the solvent front.

## 2.4. Melting point determination of isolated compound

The melting point of the crystal will be determined by introducing a tiny amount into a small capillary tube, attaching this to the stem of a thermometer centered in a heating bath. The bath slowly heated and observed for temperatures at which melting begins and is complete.

## 2.5. Spectroscopic analysis

^1^H and ^13^C NMR spectra will be recorded on Bruker Avance DMX400 FT-NMR spectrometer using TMS as internal standard, at the Department of Chemistry, Faculty of Science, Addis Ababa University, Ethiopia. Distortionless enhancements by polarization transfer (DEPT) spectra were also acquired by Bruker.

The GC-MS analysis will be conducted using ATUNE.U 5977 series Mass Hunter Acquisition Software, Agilent Technologies. The analysis condition will be; chromatographic system (7890B GC), detector (5977A MS), and column (DB-5MS, 30 m × 0.25 mm x 0.25μm). The GC experimental condition will be; inlet temperature (260 °C), injection volume (1μL), splitless mode, and total run time of 75 min. The carrier gas will be He with column flow rate 1ml/min. Oven temperature was 40 °C hold for 3 minutes, then 4 °C/min to 80°C hold for 7 min, by 4^o^C/min to 170 °C hold for 3 min, at 6 °C/min to 230^o^C hold for 4min finally, by 10°C/min to 270°C. MS experimental conditions was; ionization mode (EI), EMV mode (gain Factor), gain factor (1), transfer line temperature (280 °C), ion source temp (230°C), Quad temp (150°C), solvent delay (3 minutes), and acquisition mode (scan, 50–550 amu).

The compounds in the volatile fraction will be identified by the use of a combination of mass spectrum database search (IMS Terpene Library, NIST MS Database), relative retention time (ESO Database of EOs) and comparison of mass spectra. Quantitative analysis will be performed by peak area normalization (%) measurements (TIC, total ion count) of GC–MS chromatograms.

## 2.6. Antibacterial Activity determination

### 2.6.1. Preparation and Dilution of Test Solutions

Resazurin will be prepared at 0.015 % by dissolving 0.015 g in 100ml of Phosphate-buffered saline (PBS) , vortexed, sterilized by microfiltration and stored at 4^0^c (Elshikh *et al.*, 2016).The plant extract will be dissolved in DMSO for antibacterial activity assay. The final concentration of DMSO in the solution will be less than or equal to 2%. The stock solution of the extract/EO prepared is 5 µg/ml. This solution will be serially diluted with broth forming testing solution ranging from 2500 to 4.883 µg/ml. β-lactam antibiotics will be prepared at stock solution of 5000 µg/ml and serially diluted from 2500 to 4.883 µg/ml. Standard antibacterial agents’ stock concentration of 128 µg/ml of will be prepared and serially diluted 16 to 0.0625 µg/ml. DMSO will also be serially diluted with broth tested for antibacterial activity.

### 2.6.2. Bacterial Inoculum Preparation

The inocula will be prepared in accordance with the CLSI recommendation and standardized to 0.5 McFarland turbidity standards equivalent to 1x10^8^CFU/ml using inoculum densitometers. Then it will further be diluted to obtain the desired cell density of 5 x 10^5^CFU/ml. Briefly, an equivalent of 0.5 McFarland suspensions (1x10^8^CFU/ml) of inoculum will be prepared and then diluted 1:20 to yield 5x10^6^CFU/ml using saline media. Then, 0.02 ml of this suspension will be inoculated into 0.18 ml of broth yielding final concentration of bacteria approximately equal to 5x10^5^ CFU/ml.

### 2.6.3. Minimum Inhibitory Concentration

Minimum inhibitory concentration of the EO, fractions and controls will be determined using broth micro-dilution method (Eloff, 1998) and CLSI guideline standards and safety precautions will be strictly followed (CLSI, 2018). Stock solution of EO and fractions will be prepared and then serially diluted with broth in two-fold forming ten different solutions. The 96-well plates will be dispensed with 100 µl MHB into each well first then EO/ fractions of 100 µl were added in the first well and serially diluted up to tenth well. The excess 100 µl removed from the tenth well. Finally 100 µl of MHB mixed with inoculum will be added from the first to eleventh well. The 11^th^ well contains the broth without the intervention (fertility control) while the 12^th^ contains broth without inoculum and intervention (sterility control). The final volume in each well will be 200 µL. The bacteria will be incubated for 20-24 hours at 35-36^o^C. DMSO was evaluated as negative control while ciprofloxacin as positive control. Microbial growth will be indicated by an irreversible change of blue resazurin to pink resofurin after 20-24 hours of incubation. Minimum bactericidal concentration (MBC) will be determined by re-culturing from four consecutive well plates which have concentration equal to or less than MIC. The plates on which bacterial grow ceased was considered MBC. Measurement of optical density (OD) at 620nm were taken at 0, 2 4 18 and 24 hours. Graph of time versus OD versus concentration will be plotted at time 0h, 2h, 4h, 18h, and 20h for time-kill analysis. The plot at 18h and 20 h will be compared with resazurin assay value.

2.6.4. **Bioactivity Determination of Fractions**

Fractions collected will be regrouped based on their TLC profile reducing the fractions. The fractions will be dried and dissolved in DMSO and antibacterial activity will be determined to identify most active fraction. The percent growth inhibition of bacterium growth will be calculated according to the formula:

Percent growth inhibition =

$$100- 100*\left( OD of test well at 20h - OD of test well at 0h \right)\div(ODof control well at 20h-OD of control well at oh)$$

## 2.7. Antifungal activity

### 2.7.1. Inoculum preparation

The inoculum will be prepared from 24 hours old colonies of candida species. Approximately 1mm diameter will be suspended in 5ml saline and adjusted to 0.5 McFarland standards. The saline will be poured into the PDA slant and mixed with a sterile swab. After 10 minutes of sedimentation, the suspension will be transferred to a sterile test tube and adjusted to the 0.5McFarland standard.

### 2.7.2. Broth microdilution

Broth micro-dilution will be used to determine the minimum inhibitory concentration (MIC). Sabouraud’s Dextrose Agar (SDA) media will be used to subculture yeast following international guideline recommendation (CLSI, 2008). Each serially diluted essential oil will further diluted in a ratio of 1:50 in RPMI media (100 µl of stock solution will be added to 4.9 ml of the RPMI), giving dilutions in the range of 0.03125 to 2 mg/ml. Fluconazole and amphotericin B will be used as positive controls.

2.7.3. **Inoculum preparation**

The Inoculum will be prepared according to CLSI guidelines (CLSI, 2008b, CLSI, 2008a). The inoculum will be prepared from 24-hour-old colonies of Candida species. Approximately 1 mm diameter will be suspended in 5 ml of saline and adjusted to 0.5 McFarland Standard. The prepared suspension will be vortexed for 15 minutes and the cell density will be adjusted to 0.5 McFarland standards. This suspension will be mixed for 15 seconds with a vortex, diluted 1:50, and further diluted 1:20 with RPMI medium to obtain the two-fold test inoculum. The (twofold) inoculum will be diluted when the wells will be inoculated (CLSI, 2008b). The minimal fungicidal concentrations (MFCs) will be determined by sub-culturing fungal isolates from the well with no visible growth onto SDA plates. The inoculated plates will be incubated for 3 days at 35^o^C. MFC will be the lowest concentration of drug that yielded a negative subculture (CLSI, 2008b, Imanirampa and Alele, 2016).

2.8. **Nutritional value determination**

The protein and fat content analyses of Amessa leaf extracts will be carried out in the laboratory of Nutrition, Food Science and Technology, College of Agriculture, Hawassa University. The analyses will be made using standard procedures (AOAC 2005). The analyses of Vitamin A, B-vitamins (B6 and B12), fatty-acid profile (DHA), zinc, iron and iodine will be done in Bless-Agri Labs Co. Ltd, AA, using standard procedures.

## 2.9. Data management

Data analyses will be done using STATA. Comparison of parameters will be done using one way analysis of variance (ANOVA) and correlations between parameters will be analyzed using Pearson correlation analysis. Values will be expressed as mean ± S.D. / or proportion (percentage).

# 3. Description of the action and its effectiveness

Following the identification of priority ameessa plants for an in-depth bioassay, mimicked extraction will be done. Then, selected bioassays will be carried out. This will determine the efficacy of the ameessa plant extracts. Nutritional analysis will also be conducted after the extraction is conducted. Partitioning and compound isolation will be done guided by the bioactivities of the crude extracts.

# 4. Expected output

Precisely, the bioactivities of the selected ameessa plants will be determined. Along with this, the nutritional values of the ameessa plants will be determined by carrying out proper analytical investigation. Here, the finding will be published in the prestigious reputable international journal.

# 5. Beneficiaries

Ameessa is widely used both in rural and urban settings. Therefore, determining the bioactivities of the ameessa plants help in determining a physiological dose for regular usage. In addition, the users can decide on which plant should they rely based on the active principles and bioactivities. Therefore, the beneficiaries of this project are local community, scientific community and humankind as a whole.

# 6. Ethical Clearance

Ethical clearance will be obtained from the Ethical Review Committee of Hawassa University. Permission will be obtained from the Sidaama regional state, woreda administrators and kebele officers. Written or informed consent will be obtained from study participants after a brief explanation about the objectives of the study to the participants. Confidentiality will be kept carefully.

# 7.  Monitoring and Evaluation

Internally, the researchers will carry out the project with strict research ethics and will disclose relevant information about the conduct to the concerned quality assuring body upon reasonable request. The team will evaluate the progress of the project periodically and will document the minute of the meeting. External authority, delegates of the research and technology transfer office, may convene a relevant monitoring and evaluation meetings. The progress report and final report of the project, upon completion, will be duly communicated as per scheduled by the office. Finally, the publication will be produced following proper publication ethics.

# 8. Work plan

| **S. No** | **Main activity** | **Time/months the activities will be accomplished** | | | | | | | | | | | |
| --- | --- | --- | --- | --- | --- | --- | --- | --- | --- | --- | --- | --- | --- |
|  |  | **2022-2023** | | | | | | | | | | | |
|  |  | Dec. | Jan. | Feb. | Mar. | April | May | June | July | Aug. | Sep. | Oct. | Nov. |
| 1 | Analysis of findings of previous phases | ✓ | ✓ |  |  |  |  |  |  |  |  |  |  |
| 2 | Incorporation of ideas idea for previous finding into proposal | ✓ | ✓ |  |  |  |  |  |  |  |  |  |  |
| 3 | Stool and urine analysis |  |  | ✓ | ✓ |  |  |  |  |  |  |  |  |
| 4 |  |  |  |  | ✓ | ✓ |  |  |  |  |  |  |  |
| 5 | Antimicrobial activity study |  |  |  |  | ✓ | ✓ | ✓ |  |  |  |  |  |
| 6 | Antifungal activity study |  |  |  |  |  | ✓ | ✓ | ✓ | ✓ |  |  |  |
| 7 | Fractionation and bioactivity guided isolation |  |  |  |  |  |  |  |  | ✓ | ✓ |  |  |
| 8 | Data analysis and report write up |  |  |  |  |  |  |  |  | ✓ | ✓ | ✓ |  |
| 9 | Revision and finalizing the report |  |  |  |  |  |  |  |  |  |  | ✓ |  |
| 10 | Preparing for public dissemination |  |  |  |  |  |  |  |  |  |  |  | ✓ |
| 11 | Manuscript preparation and submission to a reputable journal |  |  |  |  |  |  |  |  |  |  |  | ✓ |

# 9.  General breakdown of the costs and costs by phases of each year

| No | Items | | Amount | Unit cost | Total cost |
| --- | --- | --- | --- | --- | --- |
| 1. | Personnel | Sample Collection | 5 person*10 day | 450 | 22,500 |
|  |  | Lab technician fee for antibacterial study | 2 person*20 day | 450 | 18,000 |
|  |  | Lab technician fee for antifungal activity study | 2 person*20 day |  | 18,000 |
|  |  | Supervisors during sample collection | 6person* 10day | 450 | 27,000 |
|  |  | Supervisors during antibacterial activity study | 6 person*20 day | 450 | 54,000 |
|  |  | Supervisors during antifungal activity study | 6 person*20 | 450 | 54,000 |
| 2 | Microtiter well plate | | 100 pcs | 500 | 50,000 |
| 3 | Culture media | |  |  | 400,000 |
| 4 | MacFarland standard solution (0.5, 1, 2, 3 & 4) | |  |  | 16750 |
| 5 | Bacterial and fungal standard strains | |  |  | 100, 000 |
| 6 | Chemicals and reagents for biochemical test | |  |  | 300,000 |
| 7 | Analysis of nutrients | | 7 | 4000 | 28,000 |
| 8 | Purchase of chemicals for nutritional analysis | | 4 | 3500 | 14,000 |
| 9 | Kits and strip | |  |  | 200,000 |
| 10 | Glove , syringe and test tube purchase | |  |  | 30,000 |
| 11 | Stationeries materials for researchers and data collectors (field bags , writing pads, pens etc )and mobile card | |  |  | 10,000 |
| 12 | Training and module preparation after result | |  |  | 50, 000 |
| 13 | 10% contingency | |  |  | 124,225 |
|  | Total | |  |  | 1,366,475 |

**References**

Aliyu, A. B., Musa, A. M., Abdullahi, M. S., Ibrahim, H., & Oyewale, A. O. (2011). Phytochemical screening and antibacterial activities of Vernonia ambigua, Vernonia blumeoides and Vernonia oocephala (asteraceae). *Acta Poloniae Pharmaceutica - Drug Research*, *68*(1), 67–73.

Banu, K. S., & Cathrine, L. (2015). General techniques involved in phytochemical analysis. *International Journal of Advanced Research in Chemical Science*, *2*(4), 25–32.

Burton, A., Smith, M., & Falkenberg, T. (2015). Building WHO ’ s global Strategy for Traditional Medicine ଝ. *European Journal of Integrative Medicine*, *7*(1), 13–15. https://doi.org/10.1016/j.eujim.2014.12.007

CLSI (2008) Reference Method for Broth Dilution Antifungal Susceptibility Testing of Yeasts. 4th ed. CLSI standard M27 ed. Wayne, PA, USA: Clinical and Laboratory Standards Inistitute

CLSI (2018) “Methods for dilution antimicrobial susceptibility tests for bacteria that grow aerobically” National Committee for Clinical Laboratory Standards.

Demma, J., Engidawork, E., & Hellman, B. (2009). Potential genotoxicity of plant extracts used in Ethiopian traditional medicine, *122*, 136–142. https://doi.org/10.1016/j.jep.2008.12.013

Eloff, Jacobus Nicolaas (1998**)** “A Sensitive and Quick Microplate Method to Determine the Minimal Inhibitory Concentration of Plant Extracts for Bacteria.” *Planta Medica* 64 (08): 711–13.

Elshikh, Mohamed, Syed Ahmed, Scott Funston, Paul Dunlop, Mark McGaw, Roger Marchant, and Ibrahim M Banat (2016) “Resazurin-Based 96-Well Plate Microdilution Method for the Determination of Minimum Inhibitory Concentration of Biosurfactants.” *Biotechnology Letters* 38 (6): 1015–19.

European Community guidelines; Council directive of 24 November 1986 on the approximation of laws, regulations and administrative provisions of the Member states regarding the protection of animals used for experimental and other scientific purposes. EEC Directive of 1986 86/609/EEC (1986).

Giday, M., Asfaw, Z., Elmqvist, T., & Woldu, Z. (2003). An ethnobotanical study of medicinal plants used by the Zay people in Ethiopia, *85*, 43–52. https://doi.org/10.1016/S0378-8741(02)00359-8

Giday, M., Teklehaymanot, T., Animut, A., & Mekonnen, Y. (2007). Medicinal plants of the Shinasha , Agew-awi and Amhara peoples in northwest Ethiopia, *110*, 516–525. https://doi.org/10.1016/j.jep.2006.10.011

Lahlou, M. (2004). Methods to study the phytochemistry and bioactivity of essential oils. *Phytotherapy Research*, *18*(6), 435–448. https://doi.org/10.1002/ptr.1465

Lulekal, E., Rondevaldova, J., Bernaskova, E., Cepkova, J., Asfaw, Z., Kelbessa, E., … Van Damme, P. (2014). Antimicrobial activity of traditional medicinal plants from Ankober District, North Shewa Zone, Amhara Region, Ethiopia. *Pharmaceutical Biology*, *52*(5), 614–620. https://doi.org/10.3109/13880209.2013.858362

Mohan, E., Suriya, S., Shanmugam, S., & Rajendran, K. (2021). Qualitative phytochemical screening of selected medicinal plants. *Journal of Drug Delivery and Therapeutics*, *11*(2), 141–144.

Nyamai, D. W., Arika, W., Ogola, P. E., Njagi, E. N. M., & Ngugi, M. P. (2016). Medicinally important phytochemicals: an untapped research avenue. *Journal of Pharmacognosy and Phytochemistry*, *4*(4), 2321–6182.

Roger, T., Pierre-Marie, M., Igor, V. K., & Patrick, V. D. (2015). Phytochemical screening and antibacterial activity of medicinal plants used to treat typhoid fever in Bamboutos division, West Cameroon. *Journal of Applied Pharmaceutical Science*, *5*(6), 034–049. https://doi.org/10.7324/JAPS.2015.50606

Sasidharan, S., Chen, Y., Saravanan, D., Sundram, K. M., & Latha, L. Y. (2011). Extraction, isolation and characterization of bioactive compounds from plants’ extracts. *African Journal of Traditional, Complementary and Alternative Medicines*, *8*(1).

Saxena, M., Saxena, J., Nema, R., Singh, D., & Gupta, A. (2013). Phytochemistry of medicinal plants. *Journal of Pharmacognosy and Phytochemistry*, *1*(6).

Teklehaymanot, T., Giday, M., Medhin, G., & Mekonnen, Y. (2007). Knowledge and use of medicinal plants by people around Debre Libanos monastery in Ethiopia. *Journal of Ethnopharmacology*. https://doi.org/10.1016/j.jep.2006.11.019

World Health Organization International Society of Hypertension Writing Group. (2003). 2003 World Health Organization (WHO)/ International Society of Hypertension (ISH) statement on management of hypertension. *Journal of Hypertension*, *21*(11), 1983–1992. <https://doi.org/10.1097/01.hjh.0000084751.37215.d2>

# THEME THREE: ACUTE AND SUB-ACUTE TOXICITY STUDIES OF THE EXTRACTS OF SELECTED AMEESSA MEDICINAL PLANTS IN ANIMAL MODELS

Serawit Deyno, PhD, School of Pharmacy, Hawassa University

Mairegu Daniel, BSc in Chemical Engineering, IoT, Hawassa University, Hawassa

Tsadiku Getachew, MD, Adaare General Hospital, Hawassa

Nigatu Tuasha, PhD in Biomedical Sciences, Hawassa CTE, Hawassa

Menfes Tadese, School of Natural Resources and Environmental Studies, Wondo Genet College of Forestry and Natural Resources, Hawassa University

Sintayehu Fekadu, B. Sc, M.Sc, PhD, School of medical laboratory technology, Hawassa University, Hawassa, SNRS

Executive Summary

**Background:** Phytochemicals are biologically active naturally occurring chemical compounds found in the medicinal plants with potential adverse health outcomes. Plants are blindly taken as safe although could adverse health outcomes. This study will investigate acute and repeated-dose toxicity studies in Ameessa Plants.

**Methods:** Acute and repeated-dose toxicity studies of decoction and EO were conducted respectively in ratsThe sample size for both acute and repeated-dose toxicity studies will be determined based on guideline recommendation from Organization for Economic Co-operation and Development (OECD_407, 2008; OECD_425, 2008). A total of 98 animals will be used. The dose levels for repeated-dose toxicity study will be determined based on the acute toxicity study findings and findings of ethnopharmacological survey

The findings will be presented in conference, public lectures and publications. Pharmaceutical product of topical preparations is expected to be produced and policy recommendations will be formulated to governments/responsible bodies for better utilization of indigenous knowledge.

# 1. Introduction

1.1. Background

In traditional usage of medicinal plants, it is the trial and error that maintained the most widely known plants to-date while the harmful once were eliminated from the oral transfer of knowledge from one generation to the other. The toxicity of the traditional medicines may be due to their selective uptake by a specific tissue or cell, or their action on the normal export of a potentially toxic metabolite from a cell to the outside (Saad et al., 2006, Moreira et al., 2018)  These days, there is a highly pressing need to test for the systemic or local toxicity of the traditionally used medicinal plants to ensure their safety (Boelsterli 2003). Among the routinely performed toxicity tests are acute and sub-acute toxicity tests; where acute toxicity test is first evaluated by measuring the median lethal dose (LD_50_) in 14 days of single dose of oral administration to the animal model (Rhiouani et al., 2008, Bhardwaj and Gupta. 2012). The sub-acute toxicity test is performed by administering the test sample daily for about three weeks to three months (Gandhar *et al.,*2013*)*. The starting doses of 5, 50, 300 or 2000 mg/kg body weight, fixed levels of expected therapeutic level, are suggested for (acute is optimal (Unuofin *et al.,* 2018).

Since there was no safety studies of any ameessa medicinal plant used among rural residents of the SNRS, the rational for the present study is to investigate acute and sub-acute toxicities of the most widely used ameessa MPs.

**Main Objective**

Investigate the safety of the most widely used ameessa plants using *in vivo* acute and sub-acute toxicity tests.

**Sample Size and Animal Allocation**

The sample size for both acute and repeated-dose toxicity studies will be determined based on guideline recommendation from Organization for Economic Co-operation and Development (OECD_407, 2008; OECD_425, 2008). A total of 98 animals will be used. The dose levels for repeated-dose toxicity study will be determined based on the acute toxicity study findings and findings of ethnopharmacological survey. Graded doses and the vehicle will be administered by oral gavage once daily for 28 consecutive days. The control groups will receive the same volume of sterile water or 4% tween 80.

The rats/mice will be allocated into experimental groups by randomization technique using lottery method and will be marked for identification purpose. The animals will be treated in rotation of every other day from control to highest dose and vice versa.

**Animals and Husbandry**

The animals will be obtained from Ethiopian Public Health Institute (EPHI). Wistar albino rats weighing between 250g – 250g will be enrolled in the study. The animals will be kept in plastic cages in environmental conditions (22–24 °C, 12 h: 12 h dark/light cycle), fed a rodent pellet diet and allowed to drink water ad libitum. All animals will be housed in standard cages in groups and acclimatized for two weeks before experiment. In acute toxicity study three female animal of the same group will be housed together while for repeated-dose toxicity study five same sex animals of the same experimental group will be housed together.

**Experimental Procedures in Acute Toxicity Study**

Acute toxicity study will be conducted and results will be interpreted before the commencement of repeated-dose toxicity study. In acute toxicity study, three rats per group housed together as recommended in OECD guideline (OECD_423, 2002). The first group will receive the vehicle and the second 300mg/kg EO at 0 hour. The third group will receive 2000mg/kg EO at 24 hour. The first two experiments will be conducted simultaneously at 0 hour while the third experiment at 48 hours and the fourth experiment at 96 hours. Rats will be fasted for 4 hours while the rats for overnight and provided with water only, and then treatment will be administered via oral gavages. Observations will be made closely for the first 4 hours, 24 h and up to 14 days after each treatment. The number of deaths within the study period will be recorded. At the end of the experiment, the animals will be sacrificed for gross anatomy investigation. The LD_50_ values will be determined based on the Globally Harmonized System of Classification and Labeling of Chemicals (GHS) (UN, 2005).

**Experimental Procedures in Repeated-Dose Toxicity Study**

Ten animals, five male and five female will be employed in accordance with the repeated dose 28-day oral toxicity study OECD guideline (OECD_407, 2008). Body weights will be measured; food consumption will be recorded and hematological/biochemical analysis as well as histopathological examination will be performed. All animals will be observed daily for mortality, signs and symptoms of toxicity. Detailed clinical observations will be recorded on day 1 (prior to test substance administration) and daily thereafter. These observations will be conducted while handling the animal and with the animal placed in good light condition. Respiratory rate, changes in skin fur, eyes and mucous membranes, occurrence of secretions and excretions, and autonomic activity (e.g., lacrimation, piloerection, pupil size, and unusual respiratory pattern) will be observed and recorded. Behavioral parameters, changes in gait, posture, and response to handling, as well as the presence of clonic or tonic movements, stereotypies (e.g., excessive grooming, repetitive circling), and bizarre behavior (e.g., self-mutilation, walking backwards) will also be observed. Body weights will be measured and recorded twice during acclimatization, at the beginning and then after every week.

At the end of the study, all animals will be fasted overnight for rats and four hours for mice (water *ad libitum*). On 29^th^ day, the animals will be weighed and the blood will be collected for hematological and biochemical analysis by cardiac puncture under anesthesia, and then the animals will be sacrificed by cervical dislocation. Finally, body organs will be harvested for detailed gross anatomy, organ weight and histopathological investigation. The animals will then be dissected and liver, kidneys, spleen, lung, and heart, will be excised and absolute weights will be determined. The relative organ weight, defined as the organ weight per 100 gram of body weight at sacrifice, will be calculated.

The specimens for histopathology will be fixed in 10% neutral buffered formalin at 18oC. About 3-4 mm thickness of each specimen of liver, kidney, and spleen will be cut and stained with hematoxylin and eosin following standard laboratory procedures. The stained sections will be examined under microscope for cellular damage or change in morphology of particular tissue.

Blood will be collected in a pre-calibrated tube containing EDTA for hematology assessments. Hematological autoanalyzer will be used to analyze hematological parameters, including red blood cell (RBC) count, white blood cell (WBC) count, hemoglobin (Hb), hematocrit (Hct), mean cell hemoglobin (MCH), mean cell volume (MCV), mean cell hemoglobin concentration (MCHC), platelet count, and others. Blood samples will be centrifuged at 3000 rpm for 10 min for biochemical analysis. The serum will be carefully aspirated with a micro-pipette into sample bottles for various biochemical assays. The clinical chemistry parameters will be assessed using serum samples analyzed under an automatic analyzer. Analytes included aspartate transaminase (AST), alanine transaminase (ALT), alkaline phosphate (ALP), total protein (TP), total cholesterol (TC), blood urea nitrogen (BUN), creatinine (Crea), total bilirubin (T-Bili), direct bilirubin (Bili-dir), albumin (Alb), triglycerides (TG), and glucose (Glu).

# 4. Description of the action and its effectiveness

Though, there is wider use of different plants as ameessa, there is no safety profile for each of those plants. Therefore, we will investigate acute and sub-acute toxicities of the selected ameessa plants. Here, animals from the service provider institutions will be purchased, acclimatized and acute and sub-acute toxicity studies will be conducted following standard experimental procedures.

# 5. Expected output

The acute and sub-acute toxicity profiles of the selected ameessa plants will be determined. Optimal dosage and use guidelines will be developed following the findings. The findings will be disseminated to wider community properly and it will be published in international reputable journals with a prestige.

# 6. Beneficiaries

Everyone benefits from this finding. A wider community may choose a better ameessa plant based on empirical evidence generated through scientific investigation. Besides, the issue related with safety will be alleviated and the wider community can utilize the ameessa plants without unforeseen threat of the unknowns..

# 7. Ethical Clearance

Ethical clearance will be obtained from the Ethical Review Committee of Hawassa University. Permission will be obtained from the Sidaama regional state, woreda administrators and kebele officers. Written or informed consent will be obtained from study participants after a brief explanation about the objectives of the study to the participants. Confidentiality will be kept carefully.

# 8.  Monitoring and Evaluation

Internally, the researchers will carry out the project with strict research ethics and will disclose relevant information about the conduct to the concerned quality assuring body upon reasonable request. The team will evaluate the progress of the project periodically and will document the minute of the meeting. External authority, delegates of the research and technology transfer office, may convene a relevant monitoring and evaluation meetings. The progress report and final report of the project, upon completion, will be duly communicated as per scheduled by the office. Finally, the publication will be produced following proper publication ethics.

# 9. General breakdown of the costs and costs by phases of each year

|  | Items | Quantity | Unit price | Total Price (ETB) |
| --- | --- | --- | --- | --- |
| 1. | Experimental animal purchasing | 100 | 80 | 800 |
| 2. | CBC | 100 | 50 | 5000 |
| 3. | Urine analysis | 100 | 40 | 4000 |
| 4. | Liver function test | 100 | 250 | 25000 |
| 5. |  |  |  |  |
| 6. | Renal function test | 100 | 80 | 8000 |
| 7. | Lipid profile | 100 | 350 | 35000 |
| 8. | Animal diet (pellet) | 100kg | 10 | 1000 |
| 9. | Hematology | 100 | 200 | 20000 |
| 10. 1 |  |  |  | 3000 |
| 11. | Research subsidiary (field trip, transportation, accommodation and perdium) |  |  | 2500 |
| 12. | Communication fee |  |  | 5000 |
| 13. | 10% contingency |  |  | 10930 |
| 14. | Total |  |  | 120230 |

# 10. Work Plan

| **S. No** | **Main activity** | **Time/months the activities will be accomplished** | | | | | | | | | | | |
| --- | --- | --- | --- | --- | --- | --- | --- | --- | --- | --- | --- | --- | --- |
|  |  | **2023-2024** | | | | | | | | | | | |
|  |  | Dec. | Jan. | Feb. | Mar. | April | May | June | July | Aug. | Sep. | Oct. | Nov. |
| 1 | Sample preparation | ✓ | ✓ |  |  |  |  |  |  |  |  |  |  |
| 2 | Animal preparation | ✓ | ✓ |  |  |  |  |  |  |  |  |  |  |
| 3 | Acute toxicity study |  |  | ✓ | ✓ |  |  |  |  |  |  |  |  |
| 4 | Repeated-dose toxicity study |  |  |  |  | ✓ | ✓ | ✓ |  |  |  |  |  |
| 5 | Data analysis and report write up |  |  |  |  |  |  |  | ✓ | ✓ | ✓ | ✓ |  |
| 6 | Revision and finalizing the report |  |  |  |  |  |  |  |  |  |  | ✓ |  |
| 7 | Preparing for public dissemination |  |  |  |  |  |  |  |  |  |  |  | ✓ |
| 8 | Manuscript preparation and submission to a reputable journal |  |  |  |  |  |  |  |  |  |  |  | ✓ |
| 9 | Final report preparation |  |  |  |  |  |  |  |  |  |  |  | ✓ |

**Reference**

B. Gandhare, S. Kavimani, B. Rajkapoor Acute and subacute toxicity study of methanolic extract of *Ceiba pentandra* (Linn.) Gaertn. on rats. Int. J. Sci. Res., 5 (2013), pp. 315-324.

B. Saad, H. Azaizeh, G. Abu-Hijleh, O. Said Safety of traditional Arab herbal medicine, Evid.-Based compl Altern. Med., 3 (2006), pp. 433-439

H. Rhiouani, J. El-Hilaly, Z.H. Israili, B. Lyoussi Acute and sub-chronic toxicity of an aqueous extract of the leaves of *Herniaria glabra* in rodents. J. Ethnopharmacol., 118 (2008), pp. 378-386

J.O. Unuofin, G.A. Otunola, A.J. Afolayan. Evaluation of acute and subacute toxicity of whole-plant aqueous extract of *Vernonia mespilifolia* less. in Wistar rats. J. Integr. Med. (2018), [10.1016/j.joim.2018.07.003](https://doi.org/10.1016/j.joim.2018.07.003).

OECD. Guidelines for the testing of chemicals. Acute Oral Toxicity-Acute Toxic Class Method (423) (2001), pp. 1-14.

OECD_407 *Test No. 407: Repeated Dose 28-day Oral Toxicity Study in Rodents*, OECD Publishing.

OECD_423 *Test No. 423: Acute Oral toxicity - Acute Toxic Class Method*, OECD Publishing.

S. Bhardwaj, D. Gupta. Study of acute, subacute and chronic toxicity test. Int. J. Adv. Res. Pharmaceu. BioSci., 2 (2012), pp. 103-129.

S.S. Moreira, L.K. Tamashiro, B.C. Jorge, P.S. Balin, S.C. HerediaVieira, G.L. Almeida, C.A.L. Cardoso, C.A.L. Kassuya, A.C. Arena Toxicological safety evaluation in acute and 28-day studies of aqueous extract from *Serjania marginata* Casar. (Sapindaceae) levels in rats J. Ethnopharmacol. (2018), [10.1016/j.jep.2018.11.024](https://doi.org/10.1016/j.jep.2018.11.024)

U.A. Boelsterli Mechanistic Toxicology.THe Molecular Basis of How Chemicals Disrupt Biological Targets (1st ed.), Taylor and Francis Inc., London and New York (2003), pp. 1-314

UN, Globally Harmonized System of Classification and Labelling of Chemicals
