# Supplementary material for: Traditional knowledge of childhood illnesses among the Sidama nation, clinical presentation and hematological findings
Source: PLoS One. 2026 Nov 10;21(11):e0340246. doi: 10.1371/journal.pone.0340246 (PMC13750600; doi:10.1371/journal.pone.0340246)
Supplement: Supplementary file 2 [file pone.0340246.s002.docx]

Date: April 10, 2026

To: The Academic Editor and Reviewers
Journal: PLOS ONE

Re: Manuscript ID: PONE-D-25-66643
Title: *Traditional Knowledge of Childhood Illnesses among the Sidama Nation, Clinical Presentation and Hematological Findings*

Dear Dr. Amur and Reviewers,

We would like to sincerely thank you for your time, constructive feedback, and positive evaluation of our manuscript. We are encouraged that all reviewers found the study technically sound, with appropriate statistical analysis and meaningful findings. We have carefully revised the manuscript in response to all comments and journal requirements.

Below, we provide a detailed, point-by-point response. All changes have been incorporated into the revised manuscript and highlighted in the tracked version.

**Responses to Journal Requirements**

**1. Formatting and style**
We have revised the manuscript to fully comply with PLOS ONE formatting and file naming requirements.

**2. Data availability statement**
We have updated the Data Availability Statement to include a **non-author institutional contact point**, as required. Research programs directorate ([rpd@hu.edu.et](mailto:rpd@hu.edu.et)) is the contact point and this is indicated in the data availability statement. The revised statement now includes the name and contact details of the relevant institutional body responsible for data access.

**3. Funding statement**
All funding-related information has been removed from the manuscript text and retained only in the Funding Statement section of the submission system.

**4. Ethics statement**
The ethics statement has been reviewed and is now included exclusively within the Methods section. It has been removed from all other sections.

**5. Figure 1 (Map and copyright issue)**
Thank you for highlighting this important issue.

- The original figure included imagery derived from proprietary sources (e.g., Google platforms), which are not compatible with the **Creative Commons Attribution (CC BY 4.0)** license required by PLOS ONE.
- But our map was created using **QGIS** which is based entirely on open-access data sources and data used for generating the map are our own
- The figure caption has been updated accordingly to reflect the data source and licensing compliance.

This ensures full compliance with CC BY 4.0 requirements.

**6. Supporting information captions**
No Supporting Information files have been added in this manuscript.

**7. Suggested citations**
No reference were suggested.

**8. Reference list**
The reference list has been thoroughly checked and corrected. No retracted articles are cited.

**Responses to Reviewers**

**Reviewer #1**

**Comment:** Minor grammatical issues and inconsistencies between numerical descriptions and tables.

**Response:**
We appreciate this observation. The manuscript has undergone thorough language editing to correct grammatical errors. Additionally, all numerical values in the text have been carefully cross-checked and aligned with the corresponding tables.

**Reviewer #2**

**Comment:** The manuscript is well written and acceptable.

**Response:**
We thank the reviewer for the positive evaluation and encouraging remarks.

**Reviewer #3**

**Comment:** Inclusion and clearer description of control groups are needed.

**Response:**
We appreciate this important suggestion. In response:

- This is a case study and the data generated were rather compared to literature values.
- We have recommended further comparative study between cases and controls (e.g., children without reported illness history and/or those treated in clinical settings).
- The Results and Discussion sections have been expanded to better contextualize our findings in relation to these groups.

These revisions improve the interpretability and scientific rigor of the manuscript.

**Conclusion**

We believe that these revisions have significantly strengthened the manuscript and addressed all concerns raised by the editor and reviewers. We sincerely hope that the revised version meets the requirements for publication in PLOS ONE.

Thank you again for your consideration.

Kind regards,
Dr. Serawit Deyno (PhD)
(On behalf of all authors)
