# Supplementary material for: Traditional knowledge of childhood illnesses among the Sidama nation, clinical presentation and hematological findings
Source: PLoS One. 2026 Nov 10;21(11):e0340246. doi: 10.1371/journal.pone.0340246 (PMC13750600; doi:10.1371/journal.pone.0340246)
Supplement: Supplementary file 3 [file pone.0340246.s003.docx]

July 28, 2026

To: The Academic Editor and Reviewers
Journal: PLOS ONE

Re: Manuscript ID: PONE-D-25-66643R1
Title: *Traditional Knowledge of Childhood Illnesses among the Sidama Nation, Clinical Presentation and Hematological Findings*

Dear Prof. Oluyinka Ajibola Iyiola

Academic Editor

PLOS One

Dear Editor,

We extend our sincere appreciation for careful review of our manuscript and for the opportunity to improve our manuscript.

We have addressed all the requested issues as follows:

**Response to Reviewer #4**

We sincerely thank the reviewer for the thorough, insightful, and constructive evaluation of our manuscript. We greatly appreciate the positive assessment of the novelty and significance of our work, particularly the recognition of its contribution to integrating Indigenous Knowledge Systems with clinical and laboratory investigations. We have carefully considered each comment and have revised the manuscript accordingly. Our point-by-point responses are provided below.

**Comment 1:** *A drastic increase in the PCT platelet index (+1015.79%) appears in the text and Table 5. It is necessary to verify whether this is a calculation error or an anomalous outlier, and to provide a more extensive commentary on such a massive spike in the discussion.*

**Response:** We sincerely thank the reviewer for carefully identifying this error. Upon re-examination of the original laboratory dataset, we found that the plateletcrit (PCT) values were inadvertently entered and calculated incorrectly during data processing, resulting in artificially inflated percentage differences and the apparent extreme increase reported in Table 5 and the Results section.

The PCT values have now been recalculated, and the corrected values have been incorporated throughout the manuscript, including Table 5 and the corresponding Results and Discussion sections. Consequently, the previously reported extreme increase (+1015.79%) has been removed and replaced with the correct findings (-6.96%).

We are grateful to the reviewer for drawing our attention to this mistake, which has substantially improved the accuracy of the manuscript.

**Comment 2:** *In Table 4, the glucose parameter lists a "Range in study: 3.4–108.8". If the unit is mg/dL, a value of 3.4 mg/dL indicates extreme, fatal hypoglycemia. The units should be verified (e.g., to check if mmol/L was intended for the minimum value).*

**Response:** We thank the reviewer for this important observation. After reviewing the original laboratory records, we confirmed that this discrepancy resulted from a transcription error in the manuscript. Specifically, two glucose measurements were mistakenly recorded in mmol/L rather than mg/dL, whereas all other glucose values were correctly expressed in mg/dL.

The affected values have now been corrected to the appropriate units, and the glucose range, descriptive statistics, and related interpretations have been updated accordingly throughout the manuscript. The revised data no longer contain the biologically implausible value identified by the reviewer.

We sincerely appreciate the reviewer for detecting this important error.

**Comment 3:** *In the laboratory results section (Table 4), the statement “A total of 12 clinical cases were investigated” appears (along with N=13 in Table 3). The authors should clearly state in the Methods section that, while the survey was conducted among 436 individuals, full clinical testing and blood sampling were performed only on a small subgroup of children (approximately 12–13 patients) who were exhibiting symptoms at the time.*

**Response:** We thank the reviewer for this valuable suggestion. We agree that the study design required clearer explanation. The manuscript has now been revised to explicitly state in the **Methods** section that the study consisted of two distinct components (line 77-82):

1. A community-based cross-sectional survey involving **436 participants** to document Indigenous knowledge, perceptions, and practices related to Feyancho.
2. A **clinical case series** involving **13 infants diagnosed with Feyancho** during the study period, in which clinical symptoms are examined and among which12 have consented for blood samples collection for haematological and biochemical analyses.

We also carefully reviewed all tables and text to ensure consistency in reporting the sample size. Clinical examinations were conducted in (N = 13) of which (N = 12) have consented for blood collection.

These revisions provide a clearer description of the study design and distinguish the epidemiological survey from the clinical investigation.

We sincerely appreciate the reviewer's positive evaluation of the scientific novelty and potential contribution of our study. We are particularly grateful for the careful review that led to the identification of the data transcription and calculation errors. Correcting these issues has substantially improved the accuracy, clarity, and overall quality of the manuscript. We believe that the revised version addresses all the reviewer's concerns and more clearly presents the findings from both the community survey and the clinical case series involving 12 infants with Feyancho.

Kind regards

Serawit Deyno (Corresponding author on behalf of all authors)
