# Supplementary material for: Traditional knowledge of childhood illnesses among the Sidama nation, clinical presentation and hematological findings
Source: PLoS One. 2026 Nov 10;21(11):e0340246. doi: 10.1371/journal.pone.0340246 (PMC13750600; doi:10.1371/journal.pone.0340246)
Supplement: Peer review history

# Peer review: Decision Letter 0

**DOI:** 10.1371/journal.pone.0340246.r001

**Authors:** Safdar Ali Amur

1 Apr 2026

Dear Dr. Deyno,

Thank you for submitting your manuscript to PLOS ONE. After careful consideration, we feel that it has merit but does not fully meet PLOS ONE’s publication criteria as it currently stands. Therefore, we invite you to submit a revised version of the manuscript that addresses the points raised during the review process.

Please submit your revised manuscript by May 16 2026 11:59PM. If you will need more time than this to complete your revisions, please reply to this message or contact the journal office at plosone@plos.org. When you're ready to submit your revision, log on to https://www.editorialmanager.com/pone/ and select the 'Submissions Needing Revision' folder to locate your manuscript file.

- A letter that responds to each point raised by the academic editor and reviewer(s). You should upload this letter as a separate file labeled 'Response to Reviewers'.
- A marked-up copy of your manuscript that highlights changes made to the original version. You should upload this as a separate file labeled 'Revised Manuscript with Track Changes'.
- An unmarked version of your revised paper without tracked changes. You should upload this as a separate file labeled 'Manuscript'.

If you would like to make changes to your financial disclosure, please include your updated statement in your cover letter. Guidelines for resubmitting your figure files are available below the reviewer comments at the end of this letter.

If applicable, we recommend that you deposit your laboratory protocols in protocols.io to enhance the reproducibility of your results. Protocols.io assigns your protocol its own identifier (DOI) so that it can be cited independently in the future. For instructions see: https://journals.plos.org/plosone/s/submission-guidelines#loc-laboratory-protocols. Additionally, PLOS ONE offers an option for publishing peer-reviewed Lab Protocol articles, which describe protocols hosted on protocols.io. Read more information on sharing protocols at https://plos.org/protocols?utm_medium=editorial-email&utm_source=authorletters&utm_campaign=protocols.

As the corresponding author, your ORCID iD is verified in the submission system and will appear in the published article. PLOS supports the use of ORCID, and we encourage all coauthors to register for an ORCID iD and use it as well. Please encourage your coauthors to verify their ORCID iD within the submission system before final acceptance, as unverified ORCID iDs will not appear in the published article. *Only*  the individual author can complete the verification step; PLOS staff *cannot*  verify ORCID iDs on behalf of authors.

We look forward to receiving your revised manuscript.

Kind regards,

Safdar Ali Amur

Academic Editor

PLOS One

Journal Requirements:

When submitting your revision, we need you to address these additional requirements.

1. Please ensure that your manuscript meets PLOS ONE's style requirements, including those for file naming. The PLOS ONE style templates can be found at

https://journals.plos.org/plosone/s/file?id=wjVg/PLOSOne_formatting_sample_main_body.pdf and

https://journals.plos.org/plosone/s/file?id=ba62/PLOSOne_formatting_sample_title_authors_affiliations.pdf.

2. In the online submission form you indicate that your data is not available for proprietary reasons and have provided a contact point for accessing this data. Please note that your current contact point is a co-author on this manuscript. According to our Data Policy, the contact point must not be an author on the manuscript and must be an institutional contact, ideally not an individual. Please revise your data statement to a non-author institutional point of contact, such as a data access or ethics committee, and send this to us via return email. Please also include contact information for the third party organization, and please include the full citation of where the data can be found.

3. Please be informed that funding information should not appear in the Acknowledgments section or other areas of your manuscript. We will only publish funding information present in the Funding Statement section of the online submission form. Please remove any funding-related text from the manuscript.

4. Your ethics statement should only appear in the Methods section of your manuscript. If your ethics statement is written in any section besides the Methods, please delete it from any other section.

5. We note that Figure 1 in your submission contain [map/satellite] images which may be copyrighted. All PLOS content is published under the Creative Commons Attribution License (CC BY 4.0), which means that the manuscript, images, and Supporting Information files will be freely available online, and any third party is permitted to access, download, copy, distribute, and use these materials in any way, even commercially, with proper attribution. For these reasons, we cannot publish previously copyrighted maps or satellite images created using proprietary data, such as Google software (Google Maps, Street View, and Earth). For more information, see our copyright guidelines: http://journals.plos.org/plosone/s/licenses-and-copyright.

We require you to either (1) present written permission from the copyright holder to publish these figures specifically under the CC BY 4.0 license, or (2) remove the figures from your submission:

a. You may seek permission from the original copyright holder of Figure 1 to publish the content specifically under the CC BY 4.0 license.

We recommend that you contact the original copyright holder with the Content Permission Form (http://journals.plos.org/plosone/s/file?id=7c09/content-permission-form.pdf) and the following text:

“I request permission for the open-access journal PLOS ONE to publish XXX under the Creative Commons Attribution License (CCAL) CC BY 4.0 (http://creativecommons.org/licenses/by/4.0/). Please be aware that this license allows unrestricted use and distribution, even commercially, by third parties. Please reply and provide explicit written permission to publish XXX under a CC BY license and complete the attached form.”

Please upload the completed Content Permission Form or other proof of granted permissions as an "Other" file with your submission.

In the figure caption of the copyrighted figure, please include the following text: “Reprinted from [ref] under a CC BY license, with permission from [name of publisher], original copyright [original copyright year].”

b. If you are unable to obtain permission from the original copyright holder to publish these figures under the CC BY 4.0 license or if the copyright holder’s requirements are incompatible with the CC BY 4.0 license, please either i) remove the figure or ii) supply a replacement figure that complies with the CC BY 4.0 license. Please check copyright information on all replacement figures and update the figure caption with source information. If applicable, please specify in the figure caption text when a figure is similar but not identical to the original image and is therefore for illustrative purposes only.

The following resources for replacing copyrighted map figures may be helpful:

USGS National Map Viewer (public domain): http://viewer.nationalmap.gov/viewer/

The Gateway to Astronaut Photography of Earth (public domain): http://eol.jsc.nasa.gov/sseop/clickmap/

Maps at the CIA (public domain): https://www.cia.gov/library/publications/the-world-factbook/index.html and https://www.cia.gov/library/publications/cia-maps-publications/index.html

NASA Earth Observatory (public domain): http://earthobservatory.nasa.gov/

Landsat: http://landsat.visibleearth.nasa.gov/

USGS EROS (Earth Resources Observatory and Science (EROS) Center) (public domain): http://eros.usgs.gov/#

Natural Earth (public domain): http://www.naturalearthdata.com/

6. Please include captions for your Supporting Information files at the end of your manuscript, and update any in-text citations to match accordingly. Please see our Supporting Information guidelines for more information: http://journals.plos.org/plosone/s/supporting-information.

7. If the reviewer comments include a recommendation to cite specific previously published works, please review and evaluate these publications to determine whether they are relevant and should be cited. There is no requirement to cite these works unless the editor has indicated otherwise.

8. Please review your reference list to ensure that it is complete and correct. If you have cited papers that have been retracted, please include the rationale for doing so in the manuscript text, or remove these references and replace them with relevant current references. Any changes to the reference list should be mentioned in the rebuttal letter that accompanies your revised manuscript. If you need to cite a retracted article, indicate the article’s retracted status in the References list and also include a citation and full reference for the retraction notice.

[Note: HTML markup is below. Please do not edit.]

Reviewers' comments:

Reviewer's Responses to Questions

**Comments to the Author**

1. Is the manuscript technically sound, and do the data support the conclusions?

Reviewer #1: Yes

Reviewer #2: Yes

Reviewer #3: Yes

**********

2. Has the statistical analysis been performed appropriately and rigorously?

Reviewer #1: Yes

Reviewer #2: Yes

Reviewer #3: Yes

**********

ade all data underlying the findings in their manuscript fully available?

The PLOS Data policy

Reviewer #1: Yes

Reviewer #2: Yes

Reviewer #3: Yes

**********

4. Is the manuscript presented in an intelligible fashion and written in standard English?

Reviewer #1: Yes

Reviewer #2: Yes

Reviewer #3: Yes

**********

Reviewer #1: MS ID PONE-D-25-66643, title "Traditional Knowledge of Childhood Illnesses among the Sidama Nation, Clinical Presentation and Hematological Findings," has valuable information. I suggest authors double-check grammar mistakes and adjust numerical values in the description according to the tables.

Reviewer #2: Manuscript "PONE-D-25-66643"; Traditional Knowledge of Childhood Illnesses among the Sidama Nation, Clinical

Presentation and Hematological Findings; is well written. The study shows new findings. It is accepted.

Reviewer #3: The authors should clearly compare their data to at least two control groups. Information about the control groups are missing. Data from controls such as infants with no history of such illnesses or with such illnesses but that were trated at the hospital or medical clinic should be included.

**********

what does this mean?). If published, this will include your full peer review and any attached files.

If you choose “no”, your identity will remain anonymous but your review may still be made public.

**Do you want your identity to be public for this peer review?**  For information about this choice, including consent withdrawal, please see our Privacy Policy

Reviewer #1: No

Reviewer #2: No

Reviewer #3: No

**********

[NOTE: If reviewer comments were submitted as an attachment file, they will be attached to this email and accessible via the submission site. Please log into your account, locate the manuscript record, and check for the action link "View Attachments". If this link does not appear, there are no attachment files.]

To ensure your figures meet our technical requirements, please review our figure guidelines: https://journals.plos.org/plosone/s/figures

You may also use PLOS’s free figure tool, NAAS, to help you prepare publication quality figures: https://journals.plos.org/plosone/s/figures#loc-tools-for-figure-preparation.

NAAS will assess whether your figures meet our technical requirements by comparing each figure against our figure specifications.

# Author comment: Author response to Decision Letter 1

**DOI:** 10.1371/journal.pone.0340246.r002

3 Jun 2026

Date: April 8, 2026

To: The Academic Editor and Reviewers

Journal: PLOS ONE

Re: Manuscript ID: PONE-D-25-66643

Title: Traditional Knowledge of Childhood Illnesses among the Sidama Nation, Clinical Presentation and Hematological Findings

Dear Dr. Amur and Reviewers,

We would like to sincerely thank you for your time, constructive feedback, and positive evaluation of our manuscript. We are encouraged that all reviewers found the study technically sound, with appropriate statistical analysis and meaningful findings. We have carefully revised the manuscript in response to all comments and journal requirements.

Below, we provide a detailed, point-by-point response. All changes have been incorporated into the revised manuscript and highlighted in the tracked version.

Responses to Journal Requirements

1. Formatting and style

We have revised the manuscript to fully comply with PLOS ONE formatting and file naming requirements.

2. Data availability statement

We have updated the Data Availability Statement to include a non-author institutional contact point, as required. Research programs directorate (rpd@hu.edu.et) is the contact point and this is indicated in the data availability statement. The revised statement now includes the name and contact details of the relevant institutional body responsible for data access.

3. Funding statement

All funding-related information has been removed from the manuscript text and retained only in the Funding Statement section of the submission system.

4. Ethics statement

The ethics statement has been reviewed and is now included exclusively within the Methods section. It has been removed from all other sections.

5. Figure 1 (Map and copyright issue)

Thank you for highlighting this important issue.

• The original figure included imagery derived from proprietary sources (e.g., Google platforms), which are not compatible with the Creative Commons Attribution (CC BY 4.0) license required by PLOS ONE.

• But our map was created using QGIS which is based entirely on open-access data sources and data used for generating the map are our own

• The figure caption has been updated accordingly to reflect the data source and licensing compliance.

This ensures full compliance with CC BY 4.0 requirements.

6. Supporting information captions

No Supporting Information files have been added in this manuscript.

7. Suggested citations

No reference were suggested.

8. Reference list

The reference list has been thoroughly checked and corrected. No retracted articles are cited.

Responses to Reviewers

Reviewer #1

Comment: Minor grammatical issues and inconsistencies between numerical descriptions and tables.

Response:

We appreciate this observation. The manuscript has undergone thorough language editing to correct grammatical errors. Additionally, all numerical values in the text have been carefully cross-checked and aligned with the corresponding tables.

Reviewer #2

Comment: The manuscript is well written and acceptable.

Response:

We thank the reviewer for the positive evaluation and encouraging remarks.

Reviewer #3

Comment: Inclusion and clearer description of control groups are needed.

Response:

We appreciate this important suggestion. In response:

• This is a case study and the data generated were rather compared to literature values.

• We have recommended further comparative study between cases and controls (e.g., children without reported illness history and/or those treated in clinical settings).

• The Results and Discussion sections have been expanded to better contextualize our findings in relation to these groups.

These revisions improve the interpretability and scientific rigor of the manuscript.

Conclusion

We believe that these revisions have significantly strengthened the manuscript and addressed all concerns raised by the editor and reviewers. We sincerely hope that the revised version meets the requirements for publication in PLOS ONE.

Thank you again for your consideration.

Kind regards,

Dr. Serawit Deyno (PhD)

(On behalf of all authors)

Attachment Submitted filename: Response to Reviewers.docx

# Peer review: Decision Letter 1

**DOI:** 10.1371/journal.pone.0340246.r003

**Authors:** Oluyinka Iyiola

9 Jul 2026

Dear Dr. Deyno,

Thank you for submitting your manuscript to PLOS ONE. After careful consideration, we feel that it has merit but does not fully meet PLOS ONE’s publication criteria as it currently stands. Therefore, we invite you to submit a revised version of the manuscript that addresses the points raised during the review process.

Please submit your revised manuscript by Aug 23 2026 11:59PM. If you will need more time than this to complete your revisions, please reply to this message or contact the journal office at plosone@plos.org. When you're ready to submit your revision, log on to https://www.editorialmanager.com/pone/ and select the 'Submissions Needing Revision' folder to locate your manuscript file.

- A letter that responds to each point raised by the academic editor and reviewer(s). You should upload this letter as a separate file labeled 'Response to Reviewers'.
- A marked-up copy of your manuscript that highlights changes made to the original version. You should upload this as a separate file labeled 'Revised Manuscript with Track Changes'.
- An unmarked version of your revised paper without tracked changes. You should upload this as a separate file labeled 'Manuscript'.

If you would like to make changes to your financial disclosure, please include your updated statement in your cover letter. Guidelines for resubmitting your figure files are available below the reviewer comments at the end of this letter.

If applicable, we recommend that you deposit your laboratory protocols in protocols.io to enhance the reproducibility of your results. Protocols.io assigns your protocol its own identifier (DOI) so that it can be cited independently in the future. For instructions see: https://journals.plos.org/plosone/s/submission-guidelines#loc-laboratory-protocols. Additionally, PLOS ONE offers an option for publishing peer-reviewed Lab Protocol articles, which describe protocols hosted on protocols.io. Read more information on sharing protocols at https://plos.org/protocols?utm_medium=editorial-email&utm_source=authorletters&utm_campaign=protocols.

As the corresponding author, your ORCID iD is verified in the submission system and will appear in the published article. PLOS supports the use of ORCID, and we encourage all coauthors to register for an ORCID iD and use it as well. Please encourage your coauthors to verify their ORCID iD within the submission system before final acceptance, as unverified ORCID iDs will not appear in the published article. *Only*  the individual author can complete the verification step; PLOS staff *cannot*  verify ORCID iDs on behalf of authors.

We look forward to receiving your revised manuscript.

Kind regards,

Prof. Oluyinka Ajibola Iyiola

Academic Editor

PLOS One

Journal Requirements:

If the reviewer comments include a recommendation to cite specific previously published works, please review and evaluate these publications to determine whether they are relevant and should be cited. There is no requirement to cite these works unless the editor has indicated otherwise.

Please review your reference list to ensure that it is complete and correct. If you have cited papers that have been retracted, please include the rationale for doing so in the manuscript text, or remove these references and replace them with relevant current references. Any changes to the reference list should be mentioned in the rebuttal letter that accompanies your revised manuscript. If you need to cite a retracted article, indicate the article’s retracted status in the References list and also include a citation and full reference for the retraction notice.

[Note: HTML markup is below. Please do not edit.]

Reviewers' comments:

Reviewer's Responses to Questions

**Comments to the Author**

Reviewer #3: All comments have been addressed

Reviewer #4: All comments have been addressed

**********

2. Is the manuscript technically sound, and do the data support the conclusions?

Reviewer #3: Yes

Reviewer #4: Yes

**********

3. Has the statistical analysis been performed appropriately and rigorously?

Reviewer #3: Yes

Reviewer #4: Yes

**********

4. Have the authors made all data underlying the findings in their manuscript fully available?

The PLOS Data policy

Reviewer #3: Yes

Reviewer #4: Yes

**********

5. Is the manuscript presented in an intelligible fashion and written in standard English?

Reviewer #3: Yes

Reviewer #4: Yes

**********

Reviewer #3: (No Response)

Reviewer #4: This study addresses a fascinating and important topic at the intersection of ethnomedicine, pediatrics, and laboratory sciences. Its aim is to document the traditional knowledge of the Sidama people (Ethiopia) regarding an infant illness known locally as "feyancho"—which comprises a mild form, *sidancho*, and a severe form, *arusicho*—and to attempt to link these beliefs with objective clinical and biochemical parameters. The work aligns perfectly with the trend of integrating Indigenous Knowledge Systems (IKS) with conventional medicine.

Combining quantitative methods (surveys of 436 households), qualitative methods (focus group discussions), and hard medical data (blood analysis) provides a comprehensive, multidimensional picture of the problem.This is the first scientific study of the "feyancho" phenomenon, which gives it significant scientific novelty.The authors rightly note that symptoms described by the community (e.g., a sunken fontanelle in *arusicho*) and laboratory findings (elevated liver enzymes, lymphocytosis, signs of microcytic anemia) suggest that the term "feyancho" encompasses real, serious medical conditions, such as chronic infections, malnutrition (kwashiorkor), or dehydration.

Despite its high potential, the text requires refinement in several areas (primarily editorial and methodological):A drastic increase in the PCT platelet index (+1015.79%) appears in the text and Table 5. It is necessary to verify whether this is a calculation error or an anomalous outlier, and to provide a more extensive commentary on such a massive spike in the discussion.In Table 4, the glucose parameter lists a "Range in study: 3.4 - 108.8". If the unit is mg/dL, a value of 3.4 mg/dL indicates extreme, fatal hypoglycemia. The units should be verified (e.g., to check if mmol/L was intended for the minimum value).In the laboratory results section (Table 4), the statement “A total of 12 clinical cases were investigated” appears (along with N=13 in Table 3). The authors should clearly state in the Methods section that, while the survey was conducted among 436 individuals, full clinical testing and blood sampling were performed only on a small subgroup of children (approximately 12–13 patients) who were actually exhibiting symptoms at the time.

The article offers significant insight and practical value. It demonstrates that traditional beliefs are not merely "myths" but represent an attempt by indigenous populations to classify real pediatric health risks. Once minor data formatting errors are corrected and the size of the laboratory group is clarified, this work will make a valuable contribution to the global health literature.

**********

what does this mean?). If published, this will include your full peer review and any attached files.

If you choose “no”, your identity will remain anonymous but your review may still be made public.

**Do you want your identity to be public for this peer review?**  For information about this choice, including consent withdrawal, please see our Privacy Policy

Reviewer #3: **Yes:**  Lutfat Abimbola Usman

Reviewer #4: No

**********

[NOTE: If reviewer comments were submitted as an attachment file, they will be attached to this email and accessible via the submission site. Please log into your account, locate the manuscript record, and check for the action link "View Attachments". If this link does not appear, there are no attachment files.]

To ensure your figures meet our technical requirements, please review our figure guidelines: https://journals.plos.org/plosone/s/figures

You may also use PLOS’s free figure tool, NAAS, to help you prepare publication quality figures: https://journals.plos.org/plosone/s/figures#loc-tools-for-figure-preparation.

NAAS will assess whether your figures meet our technical requirements by comparing each figure against our figure specifications.

# Author comment: Author response to Decision Letter 2

**DOI:** 10.1371/journal.pone.0340246.r004

28 Jul 2026

July 28, 2026

To: The Academic Editor and Reviewers

Journal: PLOS ONE

Re: Manuscript ID: PONE-D-25-66643R1

Title: Traditional Knowledge of Childhood Illnesses among the Sidama Nation, Clinical Presentation and Hematological Findings

Dear Prof. Oluyinka Ajibola Iyiola

Academic Editor

PLOS One

Dear Editor,

We extend our sincere appreciation for careful review of our manuscript and for the opportunity to improve our manuscript.

We have addressed all the requested issues as follows:

Response to Reviewer #4

We sincerely thank the reviewer for the thorough, insightful, and constructive evaluation of our manuscript. We greatly appreciate the positive assessment of the novelty and significance of our work, particularly the recognition of its contribution to integrating Indigenous Knowledge Systems with clinical and laboratory investigations. We have carefully considered each comment and have revised the manuscript accordingly. Our point-by-point responses are provided below.

Comment 1: A drastic increase in the PCT platelet index (+1015.79%) appears in the text and Table 5. It is necessary to verify whether this is a calculation error or an anomalous outlier, and to provide a more extensive commentary on such a massive spike in the discussion.

Response: We sincerely thank the reviewer for carefully identifying this error. Upon re-examination of the original laboratory dataset, we found that the plateletcrit (PCT) values were inadvertently entered and calculated incorrectly during data processing, resulting in artificially inflated percentage differences and the apparent extreme increase reported in Table 5 and the Results section.

The PCT values have now been recalculated, and the corrected values have been incorporated throughout the manuscript, including Table 5 and the corresponding Results and Discussion sections. Consequently, the previously reported extreme increase (+1015.79%) has been removed and replaced with the correct findings (-6.96%).

We are grateful to the reviewer for drawing our attention to this mistake, which has substantially improved the accuracy of the manuscript.

Comment 2: In Table 4, the glucose parameter lists a "Range in study: 3.4–108.8". If the unit is mg/dL, a value of 3.4 mg/dL indicates extreme, fatal hypoglycemia. The units should be verified (e.g., to check if mmol/L was intended for the minimum value).

Response: We thank the reviewer for this important observation. After reviewing the original laboratory records, we confirmed that this discrepancy resulted from a transcription error in the manuscript. Specifically, two glucose measurements were mistakenly recorded in mmol/L rather than mg/dL, whereas all other glucose values were correctly expressed in mg/dL.

The affected values have now been corrected to the appropriate units, and the glucose range, descriptive statistics, and related interpretations have been updated accordingly throughout the manuscript. The revised data no longer contain the biologically implausible value identified by the reviewer.

We sincerely appreciate the reviewer for detecting this important error.

Comment 3: In the laboratory results section (Table 4), the statement “A total of 12 clinical cases were investigated” appears (along with N=13 in Table 3). The authors should clearly state in the Methods section that, while the survey was conducted among 436 individuals, full clinical testing and blood sampling were performed only on a small subgroup of children (approximately 12–13 patients) who were exhibiting symptoms at the time.

Response: We thank the reviewer for this valuable suggestion. We agree that the study design required clearer explanation. The manuscript has now been revised to explicitly state in the Methods section that the study consisted of two distinct components (line 77-82):

1. A community-based cross-sectional survey involving 436 participants to document Indigenous knowledge, perceptions, and practices related to Feyancho.

2. A clinical case series involving 13 infants diagnosed with Feyancho during the study period, in which clinical symptoms are examined and among which12 have consented for blood samples collection for haematological and biochemical analyses.

We also carefully reviewed all tables and text to ensure consistency in reporting the sample size. Clinical examinations were conducted in (N = 13) of which (N = 12) have consented for blood collection.

These revisions provide a clearer description of the study design and distinguish the epidemiological survey from the clinical investigation.

We sincerely appreciate the reviewer's positive evaluation of the scientific novelty and potential contribution of our study. We are particularly grateful for the careful review that led to the identification of the data transcription and calculation errors. Correcting these issues has substantially improved the accuracy, clarity, and overall quality of the manuscript. We believe that the revised version addresses all the reviewer's concerns and more clearly presents the findings from both the community survey and the clinical case series involving 12 infants with Feyancho.

Kind regards

Serawit Deyno (Corresponding author on behalf of all authors)

Attachment Submitted filename: Response to Reviewers-III.docx

# Peer review: Decision Letter 2

**DOI:** 10.1371/journal.pone.0340246.r005

**Authors:** Gustav Komlaga

24 Sep 2026

Traditional Knowledge of Childhood Illnesses among the Sidama Nation, Clinical Presentation and Hematological Findings

PONE-D-25-66643R2

Dear Dr. Deyno

We’re pleased to inform you that your manuscript has been judged scientifically suitable for publication and will be formally accepted for publication once it meets all outstanding technical requirements.

Within one week, you’ll receive an e-mail detailing the required amendments. When these have been addressed, you’ll receive a formal acceptance letter and your manuscript will be scheduled for publication.

An invoice will be generated when your article is formally accepted. Please note, if your institution has a publishing partnership with PLOS and your article meets the relevant criteria, all or part of your publication costs will be covered. Please make sure your user information is up-to-date by logging into Editorial Manager at Editorial Manager® and clicking the ‘Update My Information' link at the top of the page. For questions related to billing, please contact billing support.

If your institution or institutions have a press office, please notify them about your upcoming paper to help maximize its impact. If they’ll be preparing press materials, please inform our press team as soon as possible -- no later than 48 hours after receiving the formal acceptance. Your manuscript will remain under strict press embargo until 2 pm Eastern Time on the date of publication. For more information, please contact onepress@plos.org.

Kind regards,

Gustav Komlaga

Academic Editor

PLOS One

Additional Editor Comments (optional):

Reviewers' comments:

Reviewer's Responses to Questions

**Comments to the Author**

Reviewer #3: All comments have been addressed

**********

2. Is the manuscript technically sound, and do the data support the conclusions?

Reviewer #3: Yes

**********

3. Has the statistical analysis been performed appropriately and rigorously?

Reviewer #3: Yes

**********

4. Have the authors made all data underlying the findings in their manuscript fully available?

The PLOS Data policy

Reviewer #3: Yes

**********

5. Is the manuscript presented in an intelligible fashion and written in standard English?

Reviewer #3: Yes

**********

Reviewer #3: The authors have made satisfactory and commendable corrections to the review comments given by the reviewer.

**********

what does this mean?). If published, this will include your full peer review and any attached files.

If you choose “no”, your identity will remain anonymous but your review may still be made public.

**Do you want your identity to be public for this peer review?**  For information about this choice, including consent withdrawal, please see our Privacy Policy

Reviewer #3: No

**********

# Editor report: Acceptance letter

**DOI:** 10.1371/journal.pone.0340246.r006

**Authors:** Gustav Komlaga

PONE-D-25-66643R2

PLOS One

Dear Dr. Deyno,

I'm pleased to inform you that your manuscript has been deemed suitable for publication in PLOS One. Congratulations! Your manuscript is now being handed over to our production team.

At this stage, our production department will prepare your paper for publication. This includes ensuring the following:

* All references, tables, and figures are properly cited

* All relevant supporting information is included in the manuscript submission,

* There are no issues that prevent the paper from being properly typeset

You will receive further instructions from the production team, including instructions on how to review your proof when it is ready. Please keep in mind that we are working through a large volume of accepted articles, so please give us a few days to review your paper and let you know the next and final steps.

Lastly, if your institution or institutions have a press office, please let them know about your upcoming paper now to help maximize its impact. If they'll be preparing press materials, please inform our press team within the next 48 hours. Your manuscript will remain under strict press embargo until 2 pm Eastern Time on the date of publication. For more information, please contact onepress@plos.org.

You will receive an invoice from PLOS for your publication fee after your manuscript has reached the completed accept phase. If you receive an email requesting payment before acceptance or for any other service, this may be a phishing scheme. Learn how to identify phishing emails and protect your accounts at https://explore.plos.org/phishing.

If we can help with anything else, please email us at customercare@plos.org.

Thank you for submitting your work to PLOS One and supporting open access.

Kind regards,

PLOS One Editorial Office Staff

on behalf of

Prof. Gustav Komlaga

Academic Editor

PLOS One
